# Optimizing the Tribological Behavior of Nanocrystalline Inconel 718 by Solid Lubricant Reinforcements

**DOI:** 10.1007/s11249-026-02198-x

**Published:** 2026-09-04

**Authors:** Manoel Kasalo, U. Pranav Nayak, Timothy MacLucas, Sebastian Suarez, Andrea Bachmaier

**Affiliations:** 1 Erich Schmid Institute of Materials Science of the Austrian Academy of Sciences, 8700 Leoben, Austria; 2 Chair of Functional Materials, Saarland University, Campus D3.3, 66123 Saarbrücken, Germany

**Keywords:** Solid lubrication friction, Self-lubricating composites, Unlubricated wear, Wear mechanisms, Nickel-based superalloys

## Abstract

Bulk metal matrix composites of nanocrystalline Inconel 718 (IN718) reinforced with various solid lubricant (SL) particles were fabricated from powder blends using a combination of colloidal mixing and high-pressure torsion (HPT). Conventional (MoS_2_, WS_2_, graphite) and modern SL (graphene nanoplatelets and functionalized carbon nanotubes) were incorporated at varying concentrations. Tribological performance was investigated under ambient conditions with a focus on running-in behavior. Linear reciprocating ball-on-disc tests were conducted against spherical Si_3_N_4_ and 100Cr6 counterbodies, with wear characterized by confocal laser microscopy and scanning electron microscopy, including qualitative chemical analysis. The friction evolution of HPT-deformed IN718 exhibited strain-dependent behavior, as evidenced by microhardness measurements. Conventional SL and graphene provided predominantly limited improvements in friction and wear. Only carbon nanotube reinforcements consistently improved tribological performance across all concentrations and counterbody materials. Best results were achieved at a content of 5 wt%, exhibiting stable self-lubricating behavior, thereby reducing the friction coefficient from 0.78 to 0.14 and the specific wear rate by an order of magnitude to ~ 10^–9^ mm^3^ N^−1^ mm^−1^. These findings highlight the potential of carbon nanotube-reinforced IN718 composites for more sustainable tribological applications under ambient conditions.

**Graphical abstract:**

**Figure:**
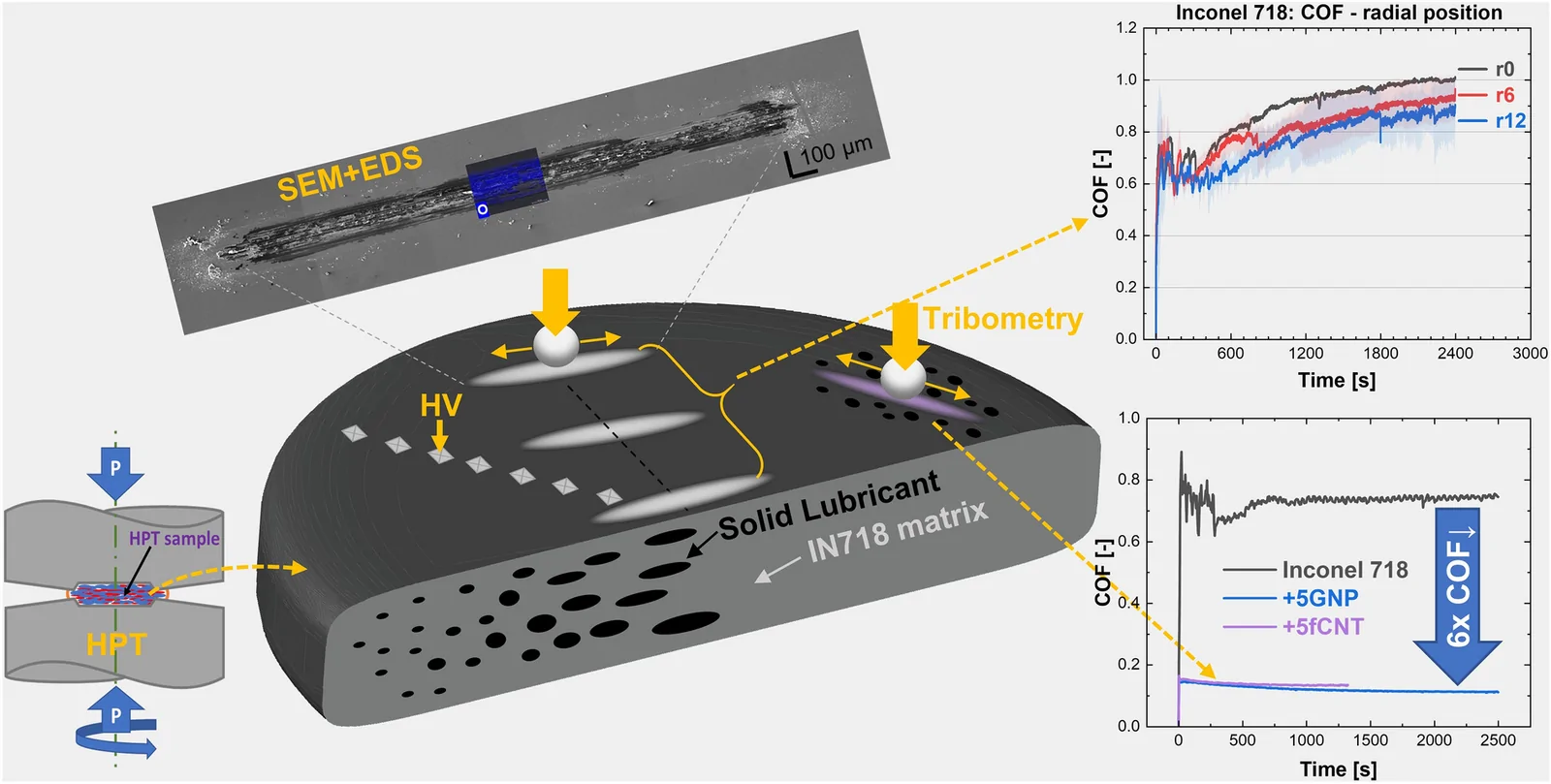

**Supplementary Information:**

The online version contains supplementary material available at https://doi.org/10.1007/s11249-026-02198-x.

## Introduction

Inconel 718 (IN718) is a widely used Ni-based superalloy for high-temperature applications due to its excellent mechanical properties and oxidation resistance. While γ′ and γ″ strengthening precipitates maintain the strength of IN718 at elevated temperatures, the oxidation resistance is attributed primarily to oxide-forming elements (e.g., Cr, Fe, and Ni), which promote the formation of passivating oxide layers that inhibit further oxygen diffusion into the alloy [1–3]. Grain refinement represents an alternative approach to improve oxidation resistance without increasing the concentration of passivating elements. It has been shown that nanocrystalline Ni–Cr alloys exhibit higher oxidation resistance than coarse-grained microstructures due to the accelerated formation of a dense and continuous protective oxide layer. With decreasing grain size, the grain-boundary volume fraction increases, providing a higher density of oxygen diffusion paths and enhancing grain-boundary diffusion of alloying elements, e.g., oxide-forming solutes such as Cr [4–6].

In tribological sliding contacts, oxidation is accelerated due to frictional heating and is generally undesirable, as it can negatively affect friction and wear behavior. Oxidation kinetics of metals under sliding conditions can be up to several orders of magnitude faster than that of static (thermal) oxidation, particularly at low temperatures [7]. Repeated oxide formation, fracture, and removal continuously expose fresh metal surfaces, thereby promoting ongoing oxidative and abrasive wear. However, tribological behavior strongly depends on system parameters, including the material properties of the counterparts and contact conditions such as load and sliding speed. While mostly steady state friction values are reported, especially at high loads and sliding speeds, there is a lack of understanding of the running-in behavior of IN718, particularly at low sliding speeds and normal loads [8]. Furthermore, the testing environment, including temperature, atmosphere, and lubrication conditions, plays a crucial role in the friction and wear behavior of IN718. For instance, Bai et al. thoroughly investigated the temperature-mediated tribo-oxidation behavior of as-received IN718 under dry sliding conditions against Si_3_N_4_ [8]. At elevated temperatures, IN718 undergoes material softening and forms a compact lubricating oxide glaze layer, reducing friction and wear. Raman analysis further indicated that multiple oxides are present on the worn IN718 surface, with Fe- and Mn-oxide species mainly detected at RT, whereas Cr_2_O_3_ becomes increasingly dominant at elevated temperature. In contrast, during sliding at room temperature (RT), a significantly higher coefficient of friction (COF) and wear rate were reported, as fragmentation of a thin and discontinuous oxide film results in three-body abrasion rather than the formation of a protective glaze [8]. This highlights the vulnerability of IN718 when operating under ambient conditions, where the absence of a stable oxide glaze necessitates alternative lubrication strategies, such as using solid lubricants (SL). Those are typically applied as coatings or dispersed as particles in a metal matrix to reduce shear stresses at the contact interface and have been shown to improve the tribological performance over a broad temperature range under extreme conditions [9–11].

In this work, the tribological performance of nanocrystalline IN718 is investigated by incorporating different types and concentrations of SL particles. Bulk metal matrix composites (MMCs) are produced from powder materials using a combination of colloidal mixing and high-pressure torsion (HPT). This severe plastic deformation (SPD) technique enables simultaneous grain refinement (nanostructuring), SL dispersion, and exceptional hardening in a single process step, as detailed in a previous work on IN718 reinforced with graphite (C) [12]. SPD is known to significantly increase defect density in graphitic structures while avoiding amorphization, as reported for HPT-deformed Ni-CNT or IN718-C composites [12, 13]. The resulting highly defective, nano-domain carbon structures may promote the formation of shearable tribofilms under sliding, thereby potentially reducing friction and wear.

The focus of this work is to study the influence of conventional SL, such as MoS_2_, WS_2_, and C, as well as novel SL, including graphene nanoplatelets (GNPs) and functionalized CNTs (fCNT), on the friction and wear behavior of IN718 after SPD. Functionalization of CNTs was required to prevent agglomeration by enhancing their interfacial bonding with the IN718 powder, thereby improving the homogeneity of the powder blend prior to HPT deformation [14]. Although the newly developed SL-reinforced IN718 composites are potential candidates for high-temperature applications, their basic tribological behavior should first be understood under mild and controlled ambient conditions. Therefore, ball-on-disc tests using linear reciprocal sliding motion were performed at low load and sliding speed to evaluate the influence of SL type and concentration, running-in behavior, and tribofilm formation without additional temperature-induced effects. This temperature was selected based on previous findings showing that the microstructure and phase stability of HPT-processed nanocrystalline IN718 composites are maintained up to ~  400 °C [15]. At higher temperatures, thermally activated processes (e.g., recovery) reduce the strain-hardening effect induced by SPD, while also approaching or exceeding the degradation temperature of the employed SL. The selected tribological conditions are not intended to reproduce a specific engineering component, but rather a generic class of slowly or intermittently actuated sliding contacts. Investigating the running-in behavior of newly developed self-lubricating composites is particularly important, as key interactions, such as surface modification, tribo-oxidation, and tribofilm formation, occur during this early sliding stage and strongly influence the subsequent friction and wear response. Wear analysis was performed using confocal laser scanning microscopy (CLSM) and scanning electron microscopy (SEM) combined with energy dispersive spectroscopy (EDS) for chemical analysis. The design of the self-lubricating IN718 composites in this work could potentially open new application fields for Ni-based superalloys in ambient environments.

## Materials and Methods

### Materials and Specimen Synthesis

Various SL-particle-reinforced IN718 composites were fabricated by combining colloidal mixing and HPT deformation starting from powder materials. To achieve this, the IN718 matrix powder was blended with 1 wt% of either transition metal dichalcogenide (TMD) or carbon nanomaterial (CNM) powders, and at 5 wt% exclusively for the carbon-based SL. For TMD powders, MoS_2_ and WS_2_ were used, whereas for CNM powders, C, GNPs, and fCNT were used. Details on powder metrics, including available particle dimensions, are provided in Supplementary Table S1. Further details on the phase constitution of present phases within HPT-processed IN718 (matrix) are provided in the Supplementary Methodology and in a previous work [16]. A prior colloidal mixing step was introduced to improve the initial homogeneity of the powder blend. Details on the mixing parameters are provided in the Supplementary Methodology and further details can be found in [12].

In the present work, large HPT discs with a diameter of 30 mm and a thickness of ~ 1.5 mm were synthesized. This enables linear reciprocal sliding at a specific radius along the tangential disc direction, allowing the study of the effect of strain gradients on friction, which are typically observed in HPT-deformed materials [17]. To obtain bulk disc-shaped specimens with a nanocrystalline IN718 matrix with finely dispersed SL, the powder blends were first consolidated via HPT. Then, the material was further deformed by torsion under a pressure of 5 GPa with a rotational speed of 0.07 min^−1^ to ensure proper co-deformation of the IN718 matrix and the embedded SL particles and to refine the IN718 matrix down to the nanoscale. Further details on the selected HPT parameters and microstructural saturation are provided in the Supplementary Methodology. Two different sets of samples were produced with different numbers of rotations. The goal was to keep the number of rotations, and thus the applied shear strain, consistent within a testing set, to ensure comparability after tribometry testing. Set 1 (IN718, + 1MoS_2_, + 1WS_2_, and +  1 C) was exposed to 10 rotations during HPT, and Set 2 (1 and 5 wt% each of C, GNP, and fCNT) to ~ 2.5 rotations. A higher number of rotations could not be applied in the second set of HPT specimens, as the high CNM concentration of 5 wt% promotes slipping between the composite and deformation tools during the deformation process.

In HPT-deformed materials, the applied shear strain (*γ*) and equivalent von Mises strain (*ε*_eq_) can be calculated according to Eq. (1) [17, 18].

1 $$\varepsilon_{{{\mathrm{eq}}}} = \frac{\gamma }{\sqrt 3 } = \frac{2\pi \cdot N \cdot r}{{t \cdot \sqrt 3 }}$$

The presented *ε*_eq_ values were calculated using the number of HPT rotations (*N* = 10 for Set 1 and *N* = 2.5 for Set 2), the respective radial position (*r*), and the final specimen thickness (*t* = 1.5 mm). This results in an *ε*_eq_ ~ 290 for set 1 and *ε*_eq_ ~ 73 for Set 2 at a radius of 12 mm for the corresponding sample thickness and number of HPT rotations mentioned before.

### Tribometry

The two specimen sets were investigated as independent but complementary studies with different tribological testing conditions. The specific testing conditions applied to each specimen set are described below. Each set included its own IN718 reference material, processed with the corresponding number of HPT rotations and tested under the same conditions as the respective composites. Therefore, direct comparisons are made only within each set.

Before tribometry testing, the HPT discs were ground plan-parallel to eliminate topographical irregularities. The test surfaces were sequentially ground up to #4000 grit, then mirror-polished with 1 µm diamond suspension, and finished with 0.05 µm colloidal silica. All micro-tribological tests were performed in a Bruker UMT TriboLab device under stable ambient conditions (temperature of 25 °C and relative humidity of 40%). Before testing, the specimens and counterbodies (CBs) were cleaned by ultrasonication in isopropanol for 10 min to remove any contaminants from their surfaces.

**Fig. 1:**
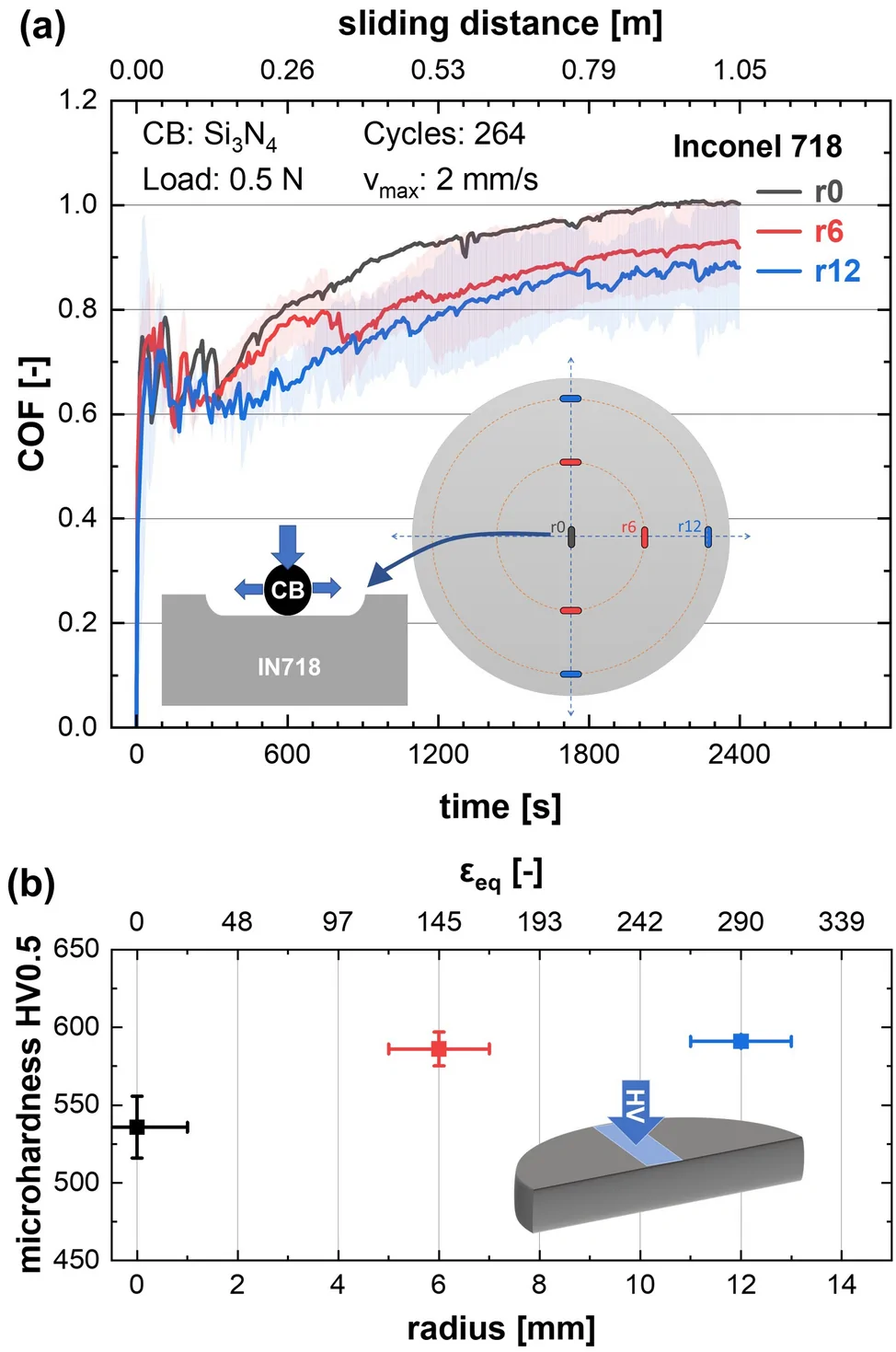
Evolution of the COF for three different radial positions (0, 6, and 12 mm) of IN718 tested against a Si_3_N_4_ CB, where the shading around the curves represents the respective standard deviation **a**. Mean microhardness values corresponding to the above-mentioned radial positions and respective equivalent von Mises strains (*ε*_eq_), calculated according to Eq. (1), are shown in **b**. The testing positions of the linear reciprocal ball-on-disc tests and microhardness measurements are shown in the corresponding schematics of the HPT discs

The first set (10 HPT rotations) compares the IN718 reference with IN718 composites reinforced with conventional SL (C, MoS_2_, and WS_2_) at the same SL fraction of 1 wt%. These were tested under a normal load of 0.5 N using a ∅6 mm Si_3_N_4_ (grade 25, 1400 HV) spherical CB, sliding against the HPT specimen in linear reciprocating motion at an average sliding speed of 0.44 mm/s, a stroke length of 2 mm for 264 cycles per wear track. This corresponds to a total sliding distance of 1.06 m or a testing time (lower speed and load) of 2400 s. To study whether the COF of the HPT-processed IN718 reference material depends on the applied shear strain and correlates directly with the radial position, three sliding tests were performed at different radial positions of 12 mm, 6 mm, and one at the disc center (0 mm) for the IN718 reference material. The load was applied along the axial specimen’s direction (perpendicular to the disc plane), while the CB was slid quasi-tangentially. The testing positions on the HPT disc are schematically shown under the COF curves in Fig. 1a. All following tests (both specimen sets) on SL-reinforced IN718 were conducted at the same radial position of 12 mm.

The second set (2.5 HPT rotations) compares the IN718 reference with IN718 composites reinforced with carbon-based SL (C, GNP, and fCNT), each at a fraction of 1 and 5 wt%. These were also tested under linear reciprocating sliding. To compare the influence of the CB material, tests were conducted at a slightly higher normal load of 1 N, using both ∅6 mm 100Cr6 steel balls (grade 28, 770 HV) and ∅6 mm Si_3_N_4_ balls (grade 25, 1400 HV). The average per cycle sliding speed was 1.13 mm/s, whereas the tests were performed for 2500 s at a stroke length of 3 mm, corresponding to 472 cycles and a sliding distance of 2.83 m, except otherwise stated. Although the average per cycle sliding speeds differed between Set 1 and Set 2 due to different acceleration settings of the lower drive, the maximum linear sliding speed reached in the middle of the wear track was kept constant at 2 mm/s for both sets. This position corresponds to the highest sliding velocity within the reciprocating stroke, where microstructural and, consequently, lubrication-related effects are expected to be most pronounced. The applied loads were selected to investigate predominantly elastic nominal Hertzian contact conditions while still generating measurable wear. The corresponding maximum Hertzian contact pressures were 577 MPa for the Si_3_N_4_/IN718 sphere-on-flat contact at 0.5 N, and 727 MPa and 637 MPa for the Si_3_N_4_/IN718 and 100Cr6/IN718 contacts at 1 N, respectively. These conditions were chosen to limit macroscopic plastic deformation and to facilitate comparison of the influence of SL type and concentration. The herein shown COF data was processed using a cycle-based peak filter, which considers the upper and lower 30% of friction force values, and averages them within a moving window corresponding to one reciprocating cycle, as detailed in Supplementary Fig. S1. Data in the vicinity of the velocity reversal points were excluded, as the sliding velocity approaches zero in these regions, leading to transient friction force fluctuations associated with the change in motion direction that are not representative of steady sliding. The COF evolution was plotted as a function of testing time to compare running-in behavior, and average COF values were additionally reported, calculated from the last 20% of the recorded data. The latter will be generally referred to as “end-of-test average COF” (*µ*_EOT_) in this work, as a steady state is not reached in each system. Running-in behavior was evaluated qualitatively from the time-dependent COF evolution, including the initial friction level, friction peaks and fluctuations, and the transition toward a more stable friction response, and compared with the characteristic running-in curve shapes described by Blau et al. [19, 20].

### Wear Analysis—Microscopy

After ball-on-disc tests, all wear tracks were analyzed using CLSM by an Olympus 4100 OLS LEXT microscope, with maximum depth and lateral resolutions of 10 and 120 nm, respectively. CLSM data were recorded at a laser wavelength of 405 nm with either a 20× or 50× objective. The specific wear rate *W*_*S*_ (mm^3^ N^−1^ mm^−1^) was used as a measure of wear performance and is defined as the volume of material removed *V* (mm^3^), normalized by the applied load, *P* (N), and the total sliding distance, *L* (mm), as given in Eq. (2).

2 $$W_{S} = \frac{V}{P \cdot L}$$

*V* was determined using the proprietary LEXT software by evaluating the complete wear track, including the end regions at the reversal points. *L* was calculated as the product of the stroke length (forward and reverse) and the number of cycles. The *W*_*S*_ values represent single measurements, with *V* evaluated once for each wear track. Accordingly, no error bars are shown in the respective figures.

Selected wear tracks were analyzed in detail by SEM (Tescan Magna) to ascertain the wear mechanisms. The micrographs were recorded by a secondary electron (SE) detector using an acceleration voltage of 5 kV and a beam current of 1 nA. Furthermore, the elemental distribution of the wear tracks was recorded with a Bruker XFlash 6|60 EDS detector to qualitatively assess chemical changes after tribometry, such as oxidation.

Vickers microhardness at different radial positions was measured only on the IN718 reference material to monitor the hardness differences between the disc center and edge. The measurements were carried out in a ZwickRoell EMCO-TEST DuraScan 70 G5 microhardness tester, where a load of 4.9 N was applied on a Vickers indenter with a dwell time of 10 s. For IN718, the indents were placed on the polished HPT disc surface, i.e., on the plane perpendicular to the axial direction, at intervals of 0.25 mm along a radial line from a radius of 0 to 14 mm. The presented IN718 mean hardness values were calculated from all available indents within the radial ranges of 0–1, 6 ± 1, and 12 ± 1 mm. For the composites, hardness measurements were performed only within the saturated outer radial region of 12 ± 1 mm, with indents placed at intervals of 0.5 mm.

## Results

### Strain-Dependent Friction Coefficient and Wear of HPT-Deformed IN718

The friction curves of IN718 against Si_3_N_4_ in Fig. 1a were averaged from three measurements (except *r* = 0 mm) at identical radial positions, whereas the standard deviation is represented as shading along the COF evolutions.

The COF evolution of IN718 is rather similar at all radial positions and can be divided into three stages. In stage 1, COF oscillates constantly around COF ~ 0.65 until ~ 400–600 s, followed by a degressive increase in COF (stage 2) between 600 and 2000 s, until approaching a steady state COF in stage 3. It is evident that COF decreases with higher radial testing position (increasing shear strain), whereas the curves at outer radii nearly overlap. For radii 0, 6, and 12 mm, *µ*_EOT_ values of 1 ± 0.01, 0.92 ± 0.01, and 0.87 ± 0.01 were obtained, respectively.

Microhardness measurements were conducted in the axial disc direction (as indicated in the schematic of the half HPT disc in Fig. 1b) at the same radial positions as the sliding tests, with values averaged over ± 1 mm around each position, as presented in Fig. 1b. The hardness increases from 536 ± 20 HV0.5 at *r* = 0 mm to 586 ± 11 HV0.5 at *r* = 6 mm and 591 ± 2 HV0.5 at *r* = 12 mm. A clear correlation is observed with increasing strain (radial position), where higher microhardness corresponds to lower COF values, both approaching saturation at higher radii.

**Fig. 2:**
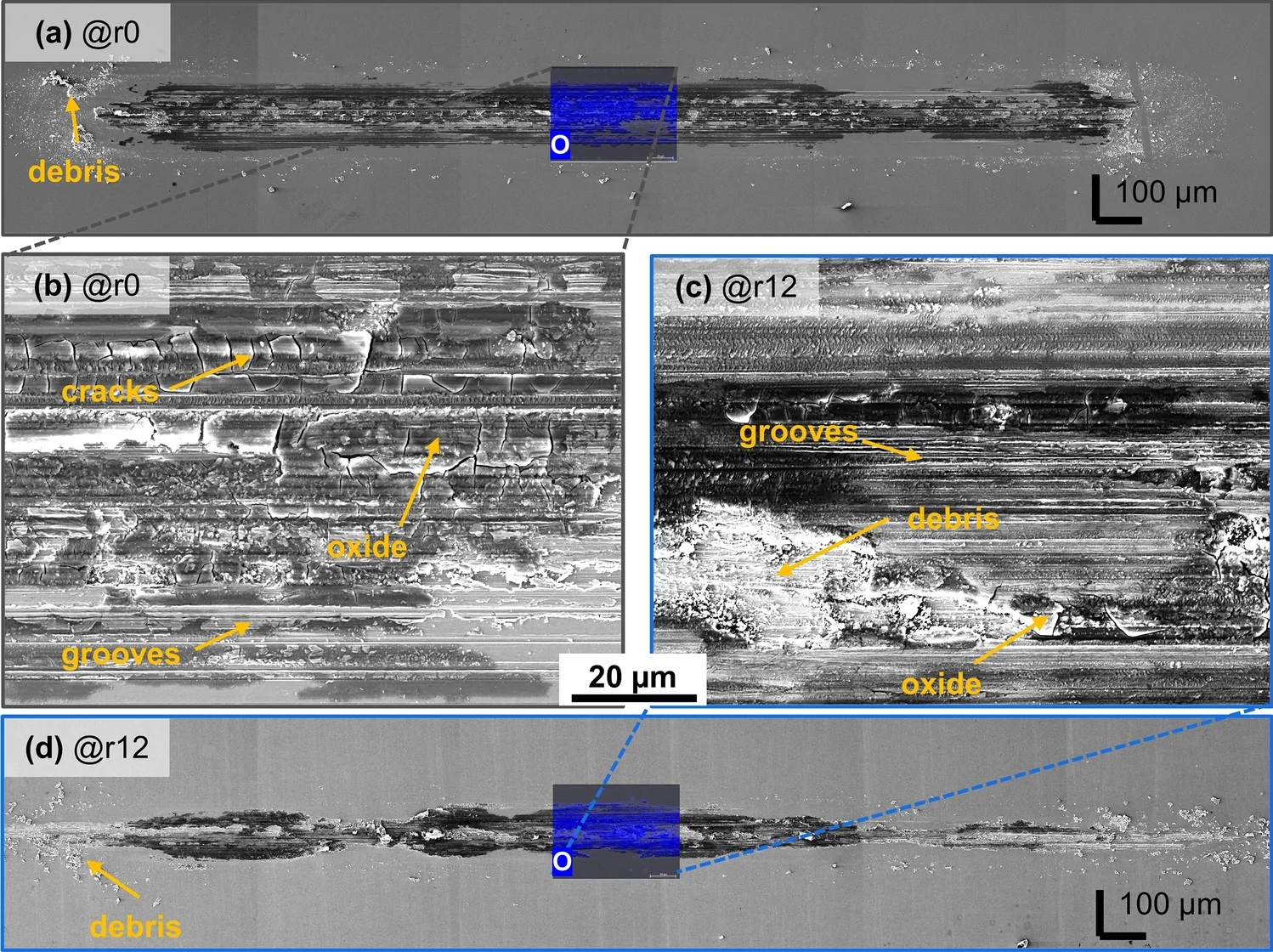
Overview SE micrographs of the wear tracks of IN718 tested at a radial position of **a** 0 mm, and **d** 12 mm. Corresponding higher magnification views of the wear tracks’ middle positions are shown in the SE micrographs in **b** and **c**, respectively. EDS maps of oxygen (blue) are overlaid with the SE micrographs of the central track regions of **a** and **d**

The wear tracks of IN718, tested at RT at radii of 0 and 12 mm are shown in the SE micrographs in Fig. 2a, and d, respectively. Compared to the disc center position, the wear track is narrower at the outer radius and exhibits a more discontinuous morphology.

Wear of HPT-deformed IN718 against Si_3_N_4_ exhibits features consistent with oxidative and abrasive wear, as shown in Fig. 2. Oxygen-enriched regions are present in all wear tracks, as evidenced by the EDS oxygen map overlays (Fig. 2a, d), and correspond to the dark areas observed in the SE micrographs. Cracked oxide layers can be identified within the wear track (Fig. 2b), together with grooves oriented parallel to the sliding direction (Fig. 2b, c). Transverse cracks might also indicate an adhesive component. Small debris particles are distributed within the wear track, while larger flake-shaped debris particles (Fig. 2a, d) are mainly located at the reversal points. In comparison, more pronounced oxide layer cracking is observed at the center (0 mm), accompanied by a broader wear track.

### Conventional Solid Lubricants as Reinforcements

**Fig. 3:**
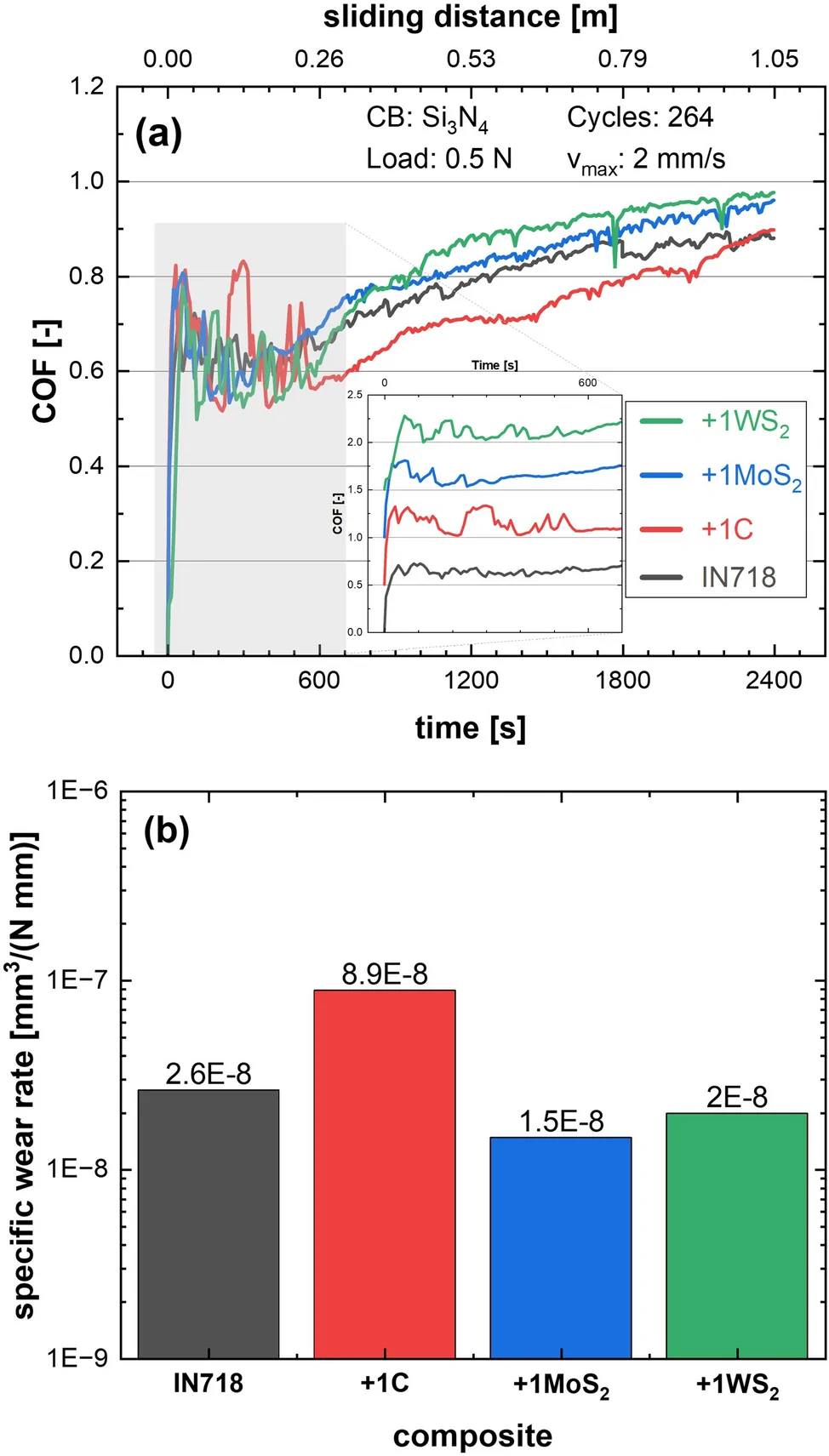
**a** Time-dependent COF evolution and **b** specific wear rate of IN718 and IN718 reinforced with 1 wt% of conventional SL particles (C, MoS_2_, and WS_2_). The values were obtained from linear reciprocal ball-on-disc tests against a Si_3_N_4_ ball, each test performed at a disc radius of 12 mm. The inset in **a** highlights the initial stage (first 700 s) of the friction curves. For better visualization, the individual curves are vertically offset by 0.5. The CLSM micrographs of the corresponding wear tracks can be found in Supplementary Fig. S2

As demonstrated in Sect. 3.1, the COF depends on the applied shear strain. Therefore, all subsequent tests in this specimen series were performed at a radial position of 12 mm, ensuring the material was tested under the same level of shear strain. Figure 3a compares the COF evolution of unreinforced IN718 with its composites reinforced with 1 wt% of conventional SL (C, MoS_2_, or WS_2_), each processed and tested under equivalent conditions.

It is evident that the COF evolutions of MoS_2_ and WS_2_ composites are consistent with that of the IN718 reference alloy, though with slightly higher COF values and higher fluctuations within the first 600 s (stage 1). The 1 wt% C composite exhibits even stronger initial fluctuations (see inset in Fig. 3a), before stabilizing at slightly lower COF values compared to the unreinforced IN718 alloy. After approximately 2200 s, the COF level converges to the same level as that of the reference material. *µ*_EOT_ values were determined as 0.87 ± 0.01 for IN718, 0.84 ± 0.03 for 1 C, 0.93 ± 0.01 for 1MoS_2_, and 0.96 ± 0.01 for 1WS_2_.

In contrast to IN718-1C, the addition of 1 wt% of TMDs did not decrease the COF. This might be associated with the differences in the lubrication performance of C and TMDs under ambient conditions, which will be discussed in Sect. 4.4.1. An opposite trend was observed for wear behavior as shown in Fig. 3b. The addition of 1 wt% C increased the specific wear rate, while 1 wt% MoS_2_ and WS_2_ reduced it marginally relative to the IN718 reference material, whereas all values remain in the same order of magnitude (~ 10^–8^ mm^3^/N^−1^ mm^−1^).

**Fig. 4:**
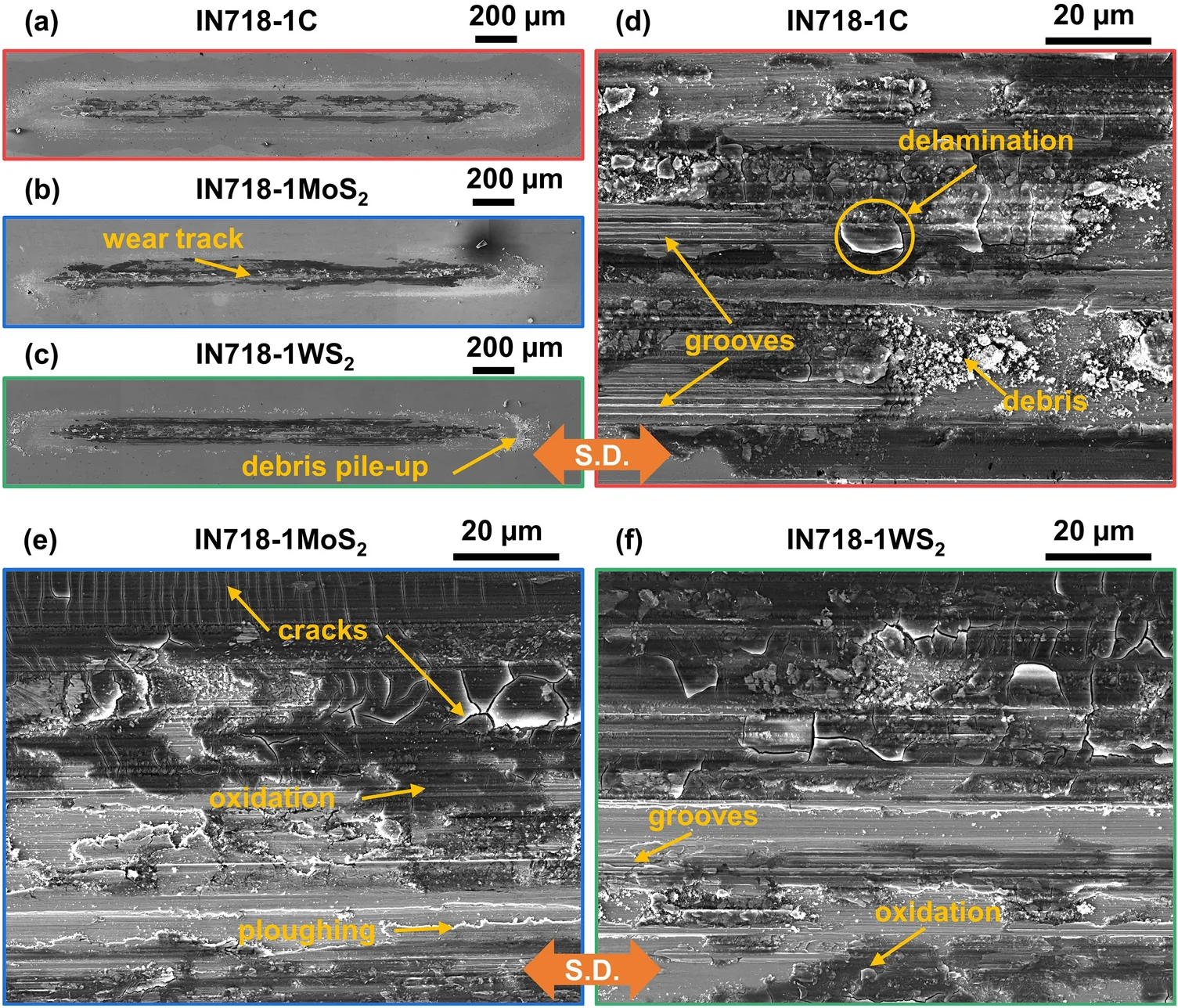
SE micrographs of the wear track overview of IN718 composites reinforced with 1 wt% of **a** graphite (C), **b** MoS_2_, and **c** WS_2_, each tested at a radius of 12 mm. A magnified representation acquired at the middle positions of the corresponding wear tracks is shown in **d**, **e**, and **f**, respectively. The sliding direction is indicated by the orange arrow “S.D”

Figure 4d–f shows that 1 wt% C, MoS_2_, and WS_2_ reinforced composites exhibit similar wear attributes as the IN718 reference alloy, as discussed before in Sect. 3.1. Oxidative and abrasive wear dominate all SL-reinforced composites. Moreover, plowing marks can be observed as a result of plastic deformation, aligned along the sliding direction as indicated in Fig. 4e. In comparison to the TMD-reinforced IN718 composites, IN718-1C exhibits indications of less oxidative wear, as implied by fewer dark regions within the wear track in the SE micrographs in Fig. 4a–c, while the wear track morphology appears more discontinuous.

### Modern Solid Lubricant Materials as Reinforcements: Carbon Nanomaterials

#### Friction Behavior for Different Solid Lubricant Types, Concentrations, and Counter bodies

**Fig. 5:**
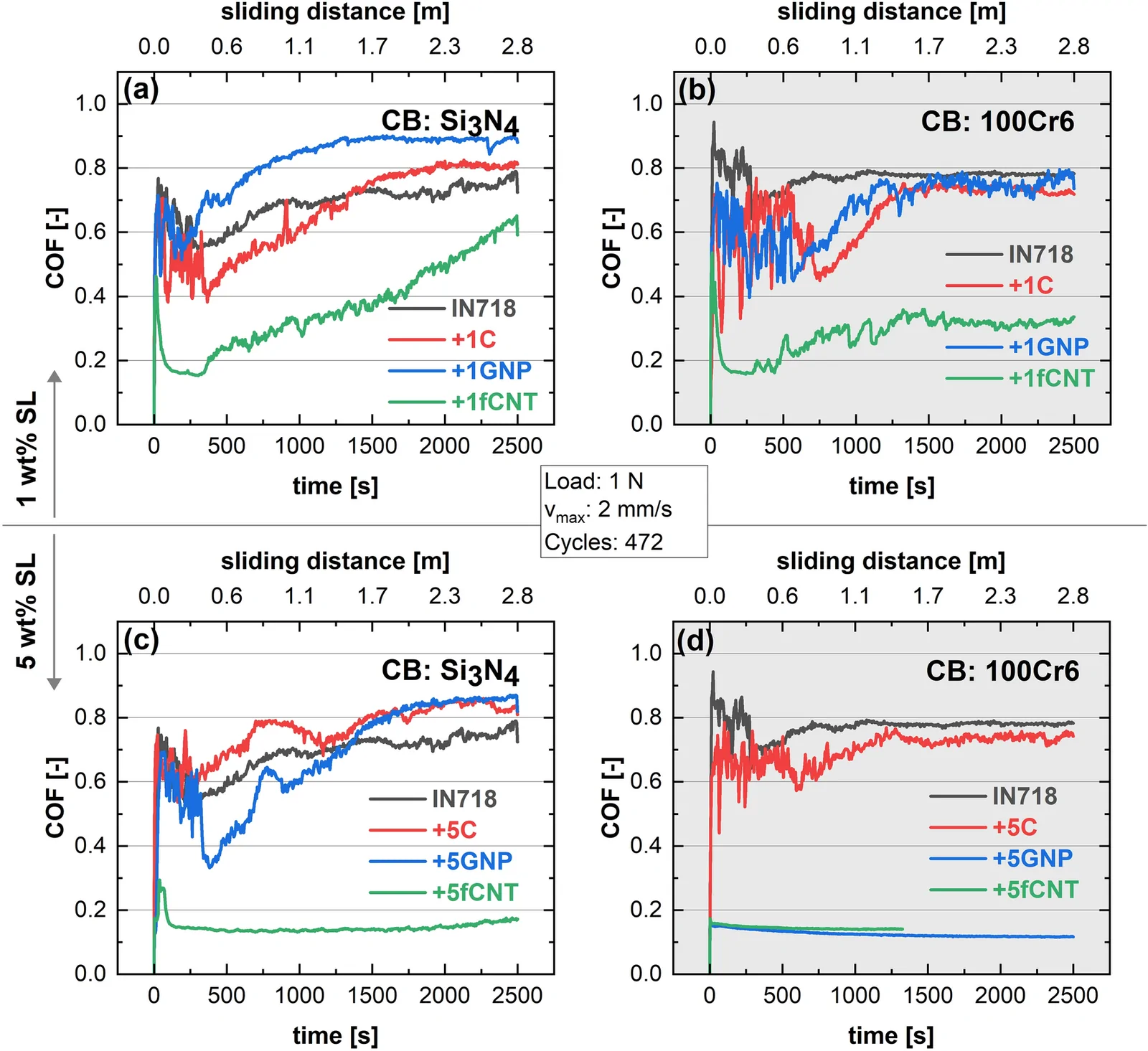
Evolution of the reciprocating COF with testing time for IN718 and IN718 reinforced with various carbon-based SL (C, GNP, and fCNT). All composites were tested under equivalent conditions and compared at similar SL concentrations of 1 wt% in **a**, **b** and of 5 wt% in **c**, **d**, each concentration tested against different CB materials (Si_3_N_4_ and 100Cr6 steel balls). All composites were exposed to 472 cycles, except for 5fCNT against 100Cr6, which was subjected to 261 cycles

The results of the previous Sect. 3.2 showed no evidence of lubrication in the TMD-reinforced IN718 composites, and mild lubrication contributions through C reinforcements. Therefore, this chapter presents the independent but complementary Set 2, which investigates the tribological behavior of IN718 composites by using C and various CNM types (GNP and fCNT) as reinforcements with different concentrations (e.g., 1 and 5 wt%), and each tested against different CB materials (i.e., Si_3_N_4_ and 100Cr6). A comparison of their COF temporal evolution is shown in Fig. 5, with all composites within Set 2 tested under equivalent conditions, except otherwise stated.

Pronounced differences are observed between the layered carbon material (C and GNP) and nanotube reinforcement types with respect to the running-in behavior. At both concentrations, the addition of C results in a temporary reduction of COF during the running-in stage. However, the COF continuously increases with sliding distance and reaches values comparable to, or even exceeding, those of unreinforced IN718. In contrast, GNPs and fCNT show a significantly stronger effect on the friction behavior. GNP-containing composites exhibit reduced COF values during the early and intermediate stages of sliding, although this effect is dependent on the SL concentration and CB material and often not sustained. The most pronounced reduction in friction is observed for fCNT-reinforced composites across all systems, which exhibit significantly lower and more stable COF values over the entire sliding distance.

Concerning the influence of SL concentration, increasing the reinforcement content from 1 to 5 wt% generally decreases friction. An exception is C, where higher contents result in increased COF values, particularly against Si_3_N_4_, whereas both CNM reinforcements benefit from higher concentrations. For GNPs, the COF reduction becomes more pronounced at 5 wt%, especially during running-in. The lowest COF (~ 0.12) of this study is observed for 5GNP, but only against 100Cr6. IN718-fCNT composites exhibit low friction levels already at 1 wt%, and at 5 wt% the COF is further reduced, showing minimal fluctuations and no significant increase with increasing sliding distance.

The tribological response is further affected by the CB material. Sliding against the 100Cr6 steel ball generally leads to lower and more stable COF values for the CNM composites than against Si_3_N_4_. This effect is particularly evident for GNP- and fCNT-containing IN718 at 5 wt%, where the COF is smoother across all stages and stabilizes at values close to 0.1 against 100Cr6, implying self-lubrication in those composites. However, the COF evolution of the 5fCNT against the ceramic CB shows a peak in stage 1 and a slight increase in friction in the last cycles, in contrast to the 100Cr6 CB tested 5fCNT composite, which stabilizes together with the 5GNP composite immediately without friction deviations at ~ 0.14 and ~ 0.12, respectively. This corresponds to a 5- to 6-fold reduction in friction compared to unreinforced IN718.

The influence of the CB is less pronounced for IN718-C, resulting in ~ 10% smaller COF values when sliding against 100Cr6 at the respective SL concentration. Higher COF levels and stronger time dependence are observed in the layered CNM composites against the ceramic CB, characterized by longer wear-in in the intermediate sliding range until approaching a steady state COF. Overall, the results demonstrate that friction reduction in SL-reinforced IN718 composites is primarily governed by the type and concentration of the SL, while the choice of the CB material further influences the friction level and the stability of lubrication.

#### Wear Behavior for Different Solid Lubricant Types, Concentrations, and Counter bodies

**Fig. 6:**
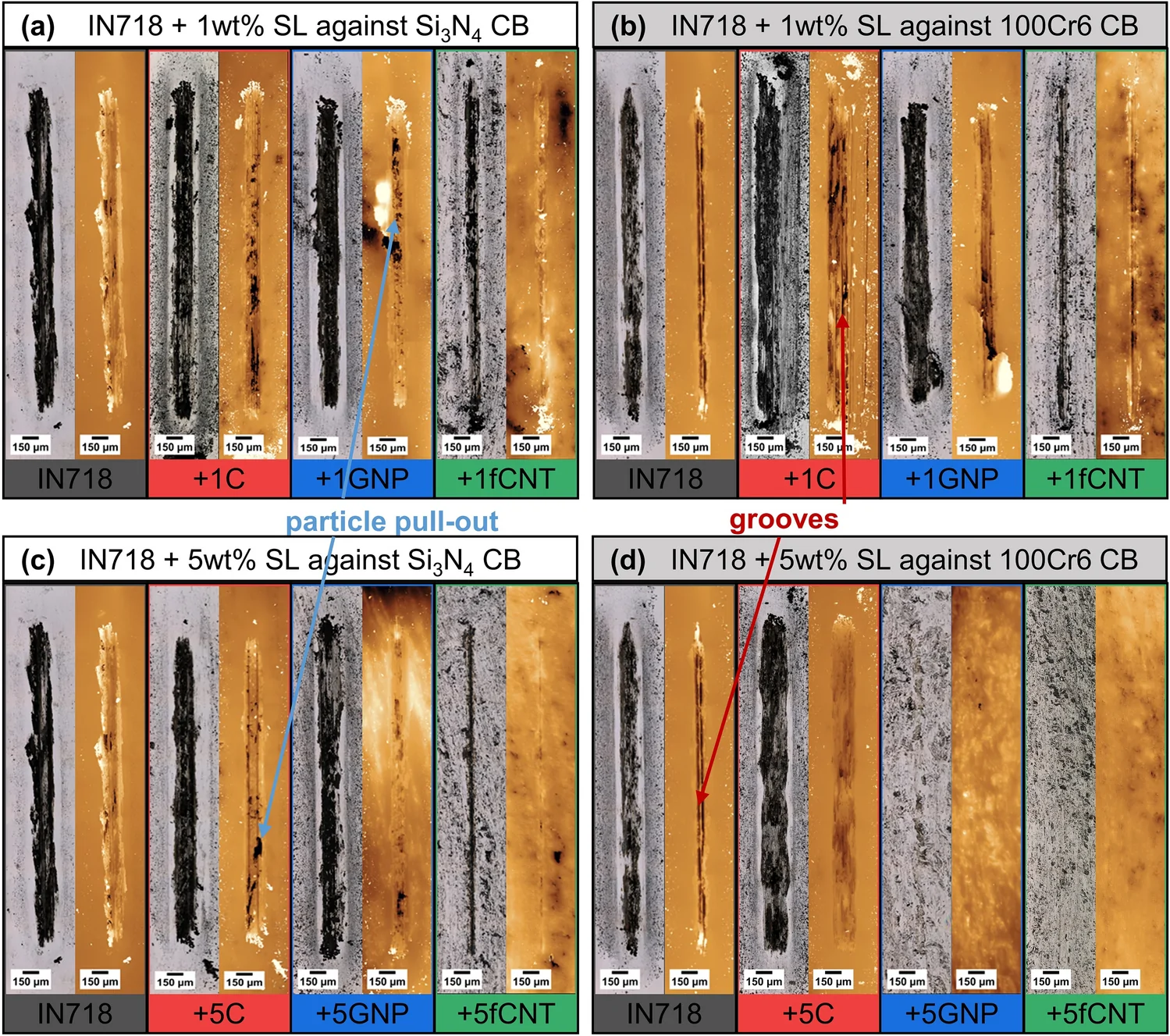
CLSM micrographs of the wear tracks of IN718 and IN718 composites reinforced with 1 wt% (**a** and **b**) or 5 wt% (**c** and **d**) of either C, GNP, or fCNT. The composites were tested either against a Si_3_N_4_ ceramic or against a hardened 100Cr6 ball and correspond to the friction curves in Fig. 5. The right-handed colored micrographs represent the respective height profiles of the left-handed optical micrographs, with lower intensities corresponding to lower surface heights. All samples were exposed to 472 cycles, except the 5fCNT against the 100Cr6 CB, which was subjected to 261 cycles

The CLSM micrographs in Fig. 6 show clear differences in wear track morphology depending on CB material, SL type, and concentration. Local height elevations within the wear scar indicate tribo-oxidation, whereas those outside the wear region are mainly attributed to debris formation and pulled-out particles, likely resulting from oxide layer fragmentation.

The unreinforced IN718 exhibits continuous grooves aligned with the sliding direction when sliding against 100Cr6, indicating pronounced microcutting (red arrows in Fig. 6b and d), whereas against Si_3_N_4,_ the wear track appears more heterogeneous and less grooved. For C-reinforced composites, wider and more irregular wear tracks are observed compared to the IN718 reference. At 1 wt% C, several dark regions are visible within the wear tracks, particularly against the ceramic CB, indicating the aforementioned particle pull-out (blue arrow in Fig. 6a and c), while distinct and numerous grooves are observed against 100Cr6, suggesting greater microcutting. Increasing the C content to 5 wt% does not suppress these features or, in general, wear.

GNP-reinforced composites show similar behavior to IN718-C for most conditions. However, at 5 wt% against 100Cr6 (Fig. 6d), wear was not detectable at this magnification, consistent with the COF evolution in Fig. 5, showing a *µ*_EOT_ ~ 0.12, indicating self-lubricating behavior.

The most pronounced wear reduction is generally observed for the fCNT-reinforced composites. At 1 wt% fCNT, wear tracks are narrow and smooth for both CB materials, with only minor local variations, while at 5 wt% fCNT, the tracks exhibit negligible topological contrast at this magnification. Only mild scratches are noticeable at a higher magnification of IN718-5fCNT against the 100Cr6 CB, which is represented in Supplementary Fig. S3.

Furthermore, the CB affects the wear characteristics. Sliding against 100Cr6 generally results in deep valleys observed in the wear track when sliding against the 100Cr6 CB, indicating severe material removal, likely associated with abrasive wear (grooves in Fig. 6). In contrast, for nanocarbon-reinforced and self-lubricating composites, especially those containing fCNT at 5 wt%, the influence of the CB is less pronounced, as wear profiles are consistently shallow and smooth for both CB materials. This trend agrees with the friction results of Fig. 5, as higher and less stable COF values are mainly observed for IN718 and C-reinforced composites against 100Cr6, whereas nanocarbon-reinforced MMCs show low and largely CB-independent COF values.

**Fig. 7:**
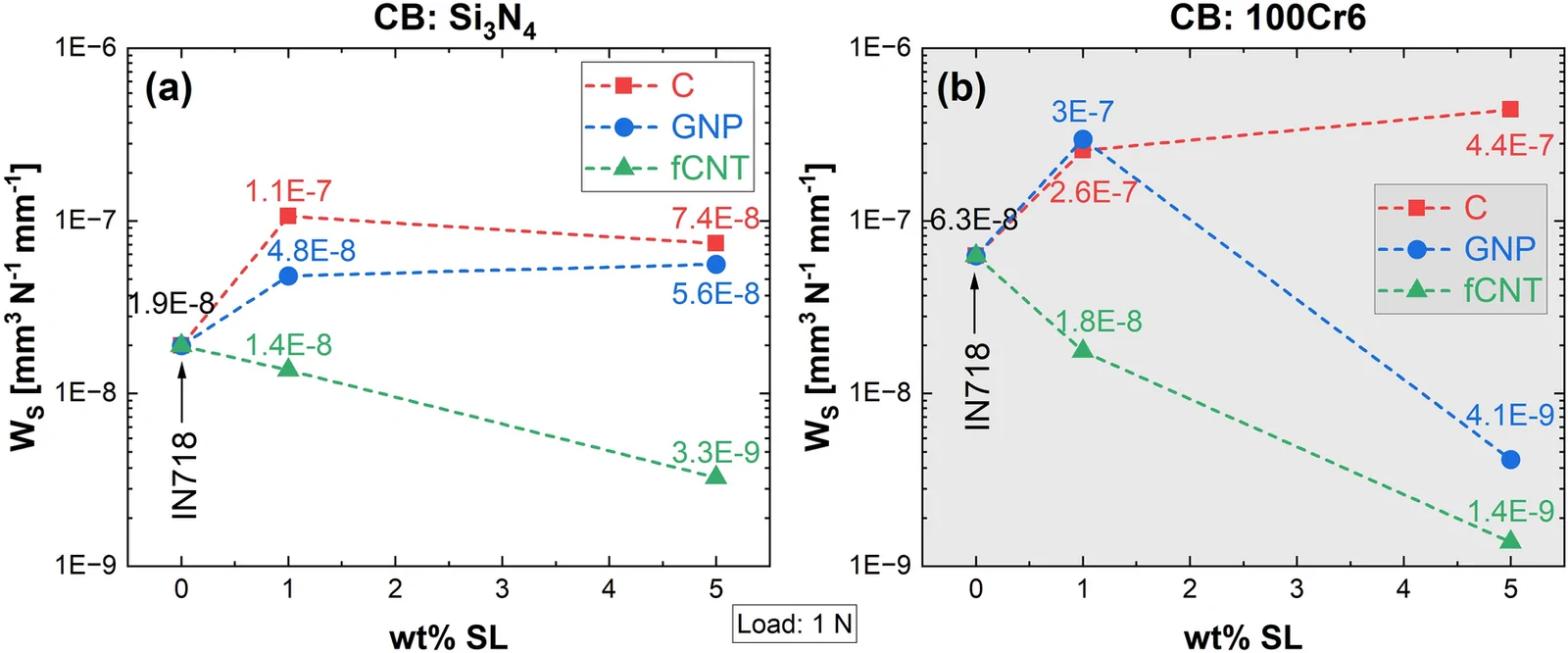
Specific wear rate (*W*_*S*_) of IN718 and IN718 composites reinforced with C, GNP, or fCNT for SL concentrations up to 5 wt%. The composites were tested either against a Si_3_N_4_ ceramic **a** or a hardened 100Cr6 ball **b** and correspond to the wear tracks in Fig. 6. The specific wear rate values were calculated using Eq. (2)

Figure 7 summarizes the specific wear rates of C-, GNP, and fCNT-reinforced IN718 MMCs determined from the wear tracks in Fig. 6 according to Eq. (2). The IN718 reference material exhibits a higher wear rate (in the range of 10^–8^ mm^3^ N^−1^ mm^−1^) when sliding against 100Cr6, which correlates with the deeper grooves observed in Fig. 6b. Only fCNT-reinforced composites exhibit reduced wear, independent of the CB, whereas C- and GNP-reinforcements (except at 5wt% against 100Cr6) increase the specific wear rate of IN718 composites, indicating a transition toward severe wear. For both CB, IN718-C exhibits the highest wear rates (up to ~ 10^–7^ mm^3^ N^−1^ mm^−1^), even higher than the IN718 reference material, and shows no beneficial effect across the investigated concentrations. IN718-GNP composites exhibit a pronounced dependence on both concentration and CB. While wear increases at low concentrations, a significant reduction is observed at higher content when sliding against 100Cr6, whereas no comparable improvement is found for Si_3_N_4_. In contrast, fCNTs consistently reduce the wear rate (down to ~ 10^–9^ mm^3^ N^−1^ mm^−1^) of IN718 with increasing concentration for both CB, yielding the lowest wear at the highest content.

**Fig. 8:**
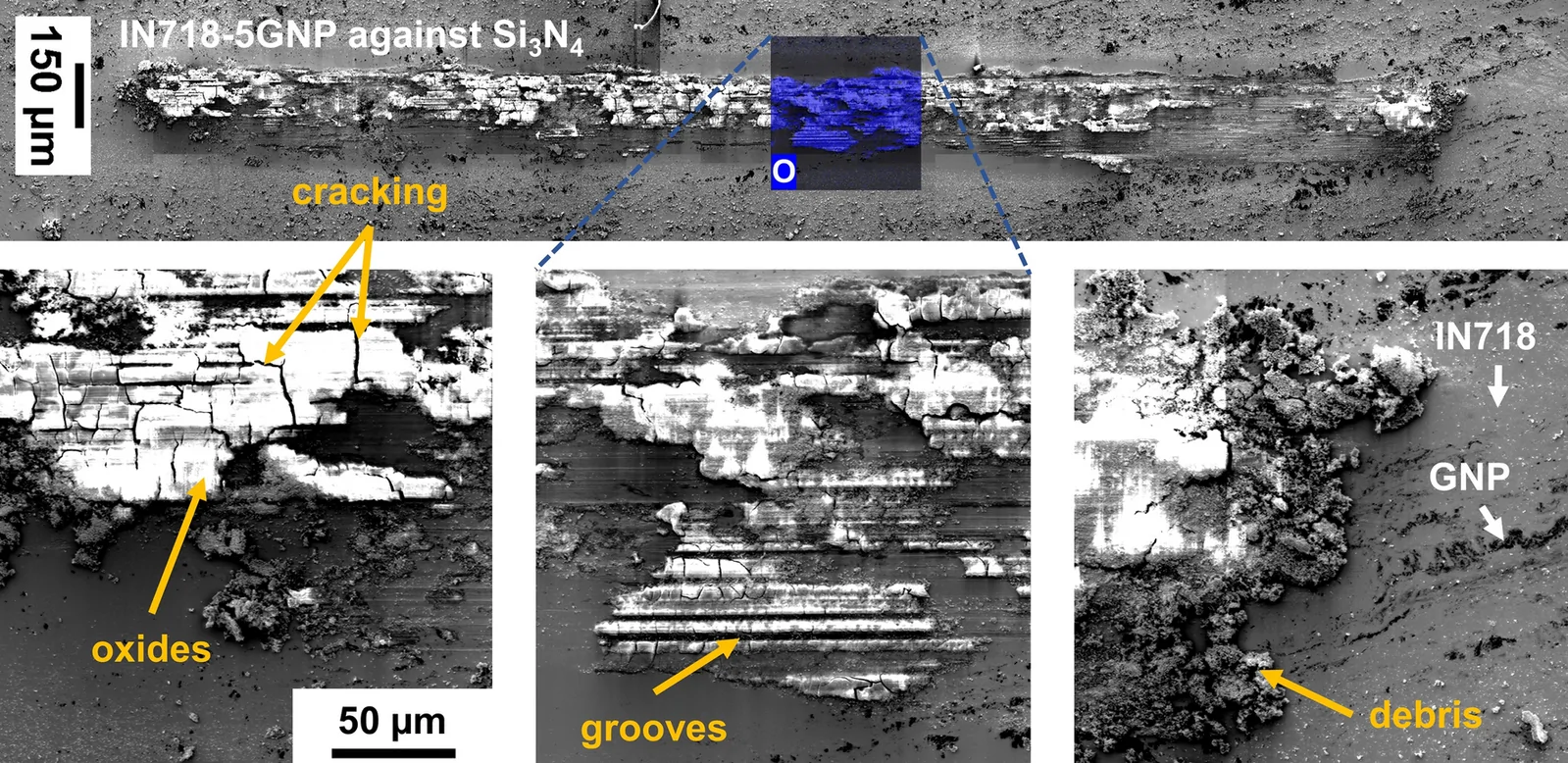
SE micrographs of the IN718-5GNP wear track tested against Si_3_N_4_ at a radial position of 12 mm. An overview of the whole wear scar (upper micrograph) is shown in the upper micrograph, including an oxygen EDS map at the center position, and detailed views of the center and reversal points of the wear track are presented in the lower micrographs

As aforementioned, no distinct height differences are observed in the Si_3_N_4_ tested GNP composites, although wear scars are evident in Fig. 6. This is attributed to the formation of an oxide layer that is not sufficiently worn off to see height differences, as evident in the micrographs of Fig. 8. Basically, similar wear attributes can be observed as already discussed for the composites before.

**Fig. 9:**
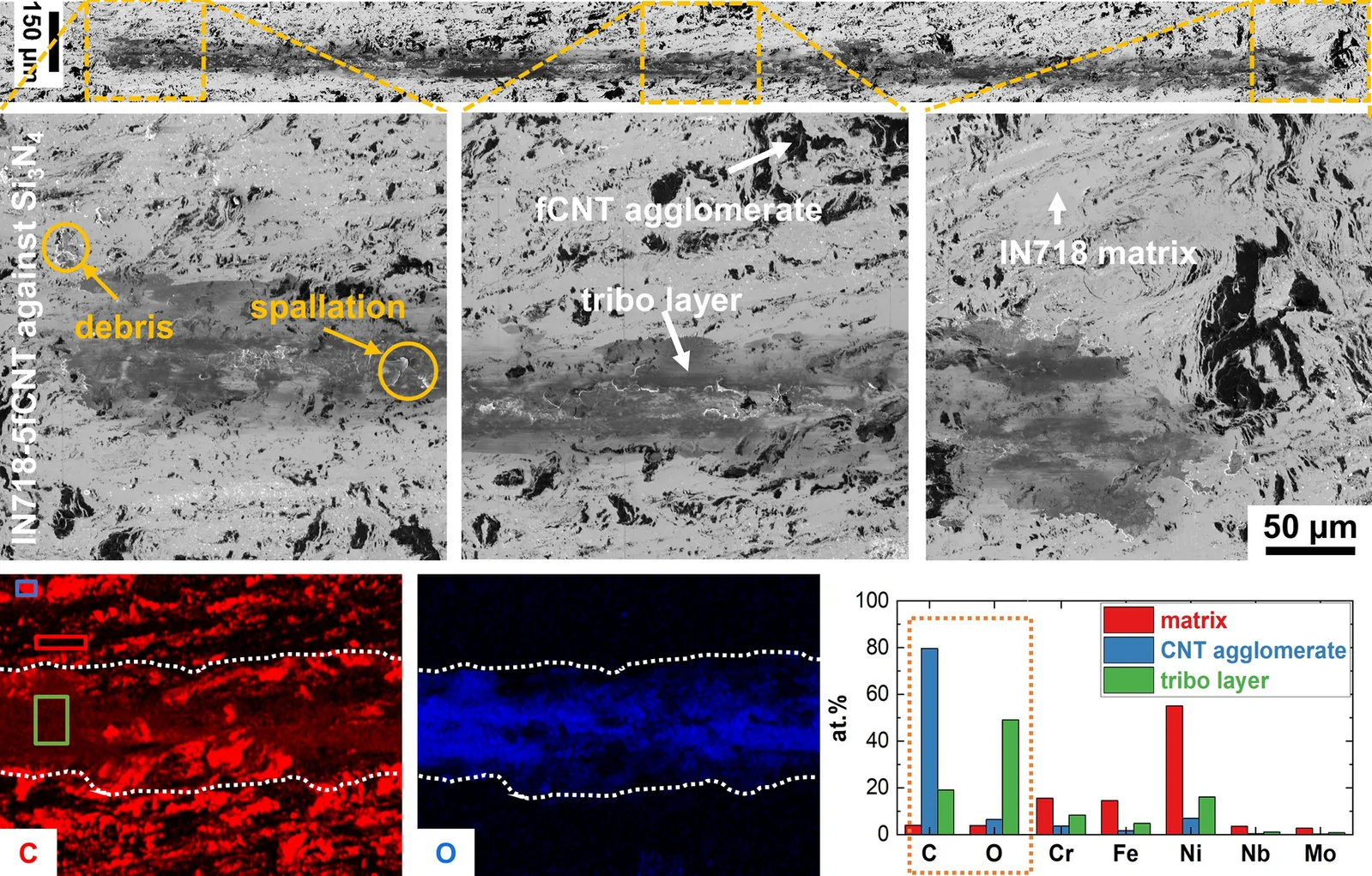
SE micrographs and elemental EDS maps of carbon (C) and oxygen (O) of the IN718-5fCNT composite’s wear track tested against Si_3_N_4_. The concentrations of the elements are compared for three different regions of interest, indicated by rectangles in the carbon map: CNT agglomerate (blue rectangle), the matrix (red rectangle), and within the tribolayer (green rectangle). The EDS maps were acquired at the middle position of the wear track

Due to significantly reduced friction and wear observed for IN718-5fCNT, a detailed wear analysis was performed exemplarily on the 5fCNT composite tested against Si_3_N_4_ using SEM and EDS, as shown in Fig. 9. The wear track topography observed in the SE micrographs is consistent with the CLSM micrographs (Fig. 6c), showing no significant height differences between the tested area and the surrounding matrix.

Furthermore, a tribolayer can be clearly observed within the wear track, appearing darker in contrast than the surrounding IN718 matrix. EDS analysis in Fig. 9 shows that this tribolayer (green rectangle) contains a higher carbon concentration than the IN718 matrix (red rectangle), while remaining lower than that of the CNT agglomerates (blue rectangle), as highlighted by the carbon EDS map and the corresponding bar chart. This indicates the formation of a carbon-rich, self-lubricating layer during sliding against Si_3_N_4_, which likely contributes to the observed reduction in friction and wear. Simultaneously, the wear track exhibits an increased oxygen content compared to the matrix, indicating that tribo-oxidation occurs concurrently with lubrication. This results in minor spallation and debris formation, as marked in the left SE micrograph of the reversal point. However, the extent of oxidation is significantly lower than for the IN718 reference alloy or for composites that do not exhibit self-lubrication, suggesting that the formation of abrasive third-body oxide particles is largely suppressed under these conditions.

Overall, IN718 shows a clear dependence of the radial testing position on microhardness, indicating strain-dependent behavior. When sliding against Si_3_N_4_, wear in IN718 is dominated by oxidative and abrasive mechanisms, which were also observed in composites that do not exhibit self-lubricating behavior. At comparable strain levels, IN718 composites reinforced with 1 wt% of conventional SL (C, MoS_2_, and WS_2_) show friction and wear behavior similar to the IN718 reference. Among these, only IN718-C exhibits slightly reduced friction but increased wear, indicating a distinct behavior of C under controlled ambient dry sliding conditions compared to TMD reinforcements, which is discussed in Sect. 4.4.1. Increasing the C content does not improve friction or wear, regardless of the CB material. In general, sliding against 100Cr6 promotes more pronounced cutting wear than against Si_3_N_4_. In contrast, modern SL, such as GNP and fCNT, show significantly different behavior, as discussed in Sect. 4.4.2. IN718-fCNT composites exhibit a consistent and significant reduction in both COF and wear rate across all concentrations and CB materials, with clear indications of self-lubricating characteristics at 5 wt%. GNP-reinforced composites predominantly behave consistently with IN718-C, except for IN718-5GNP against 100Cr6, where indications of self-lubrication were also observed.

### Strain-Dependent Microhardness of Solid Lubricant-Reinforced IN718

**Fig. 10:**
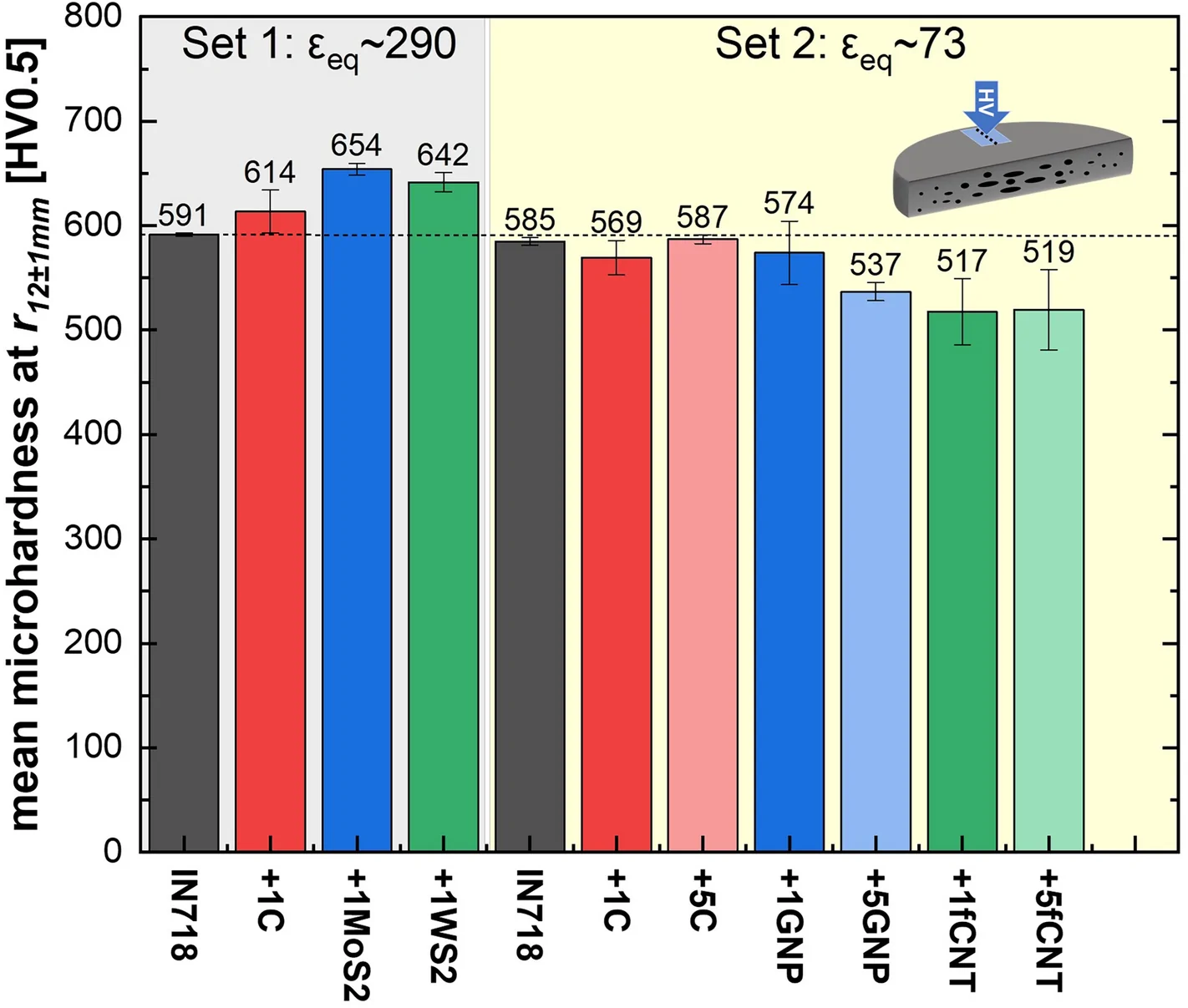
Mean microhardness at the radial position around 12 mm for both specimen sets. Set 1 includes IN718 and its composites reinforced with 1 wt% of conventional SL (C, MoS_2_, and WS_2_), subjected to higher strain (*ɛ*_eq_ ~ 290), and Set 2 was subjected to lower strain (*ɛ*_eq_ ~ 73) and includes IN718 and its composites reinforced with either 1 or 5 wt% of carbon-based SL (C, GNP, and fCNT). The tested radial position is indicated schematically on the HPT disc

Figure 10 shows the mean microhardness of all tested composites at the radial position around 12 mm, corresponding to the tribological testing position of the MMCs. Although specimens Set 1 and Set 2 are considered separately with respect to their tribological behavior, their hardness values are shown together for comparison. The composites from Set 1 (1 wt% C, MoS_2_, and WS_2_) exhibit higher hardness than the IN718 reference material, indicating a reinforcement effect through the embedded SL particles. The TMD-reinforced composites show a stronger hardness increase than the C-reinforced composite, with a relative increase of up to approximately 10%.

Set 2 (C, GNP, and fCNT) was exposed to an approximately four times lower equivalent strain than Set 1, with *ε*_eq_ ~ 73 and *ε*_eq_ ~ 290 at *r* = 12 mm, respectively. The IN718 reference from Set 2 reaches 585 HV0.5, while the carbon-based composites show comparable or lower hardness values ranging from 517 to 587 HV0.5 for all SL fractions. No clear trend is observed with increasing SL fraction within the same SL type. However, the SL type appears to influence the hardness. While the IN718-C and IN718-GNP composites show comparable hardness values, the IN718-fCNT MMCs exhibit clearly lower hardness, reaching approximately 13% lower values than the corresponding IN718 reference material.

Comparing the hardness levels of both sets shows that the lower applied strain generally results in lower hardness values for the composites. However, only a marginally lower hardness is observed for the less strained IN718 reference material from Set 2, and both IN718 references exhibit small standard deviations. In contrast, the mean hardness of IN718-1C from Set 2 is approximately 8% lower than that of the same composite from Set 1.

## Discussion

### Strain-Dependent Friction and Wear of IN718

**Fig. 11:**
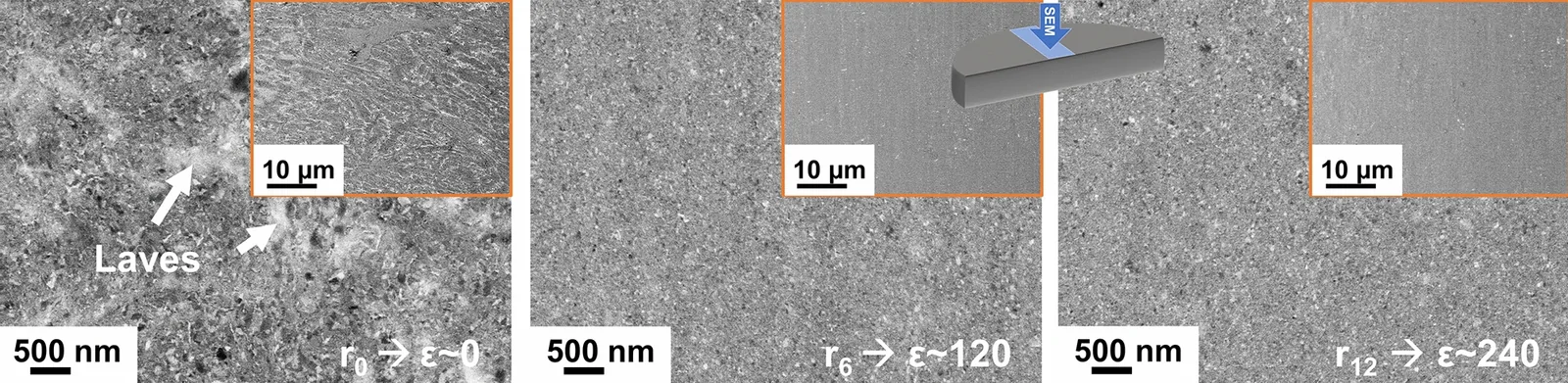
Microstructure (backscattered electron micrographs) of as-deformed IN718 (10 rotations), acquired in the axial direction at radial positions of 0, 6, and 12 mm, corresponding to equivalent strains of 0, 120, and 240%, respectively. The acquisition direction is indicated in the schematic. The respective insets show the microstructure at lower magnification

The friction coefficient of IN718 against Si_3_N_4_ (Fig. 1a) shows a clear dependence on the radial testing position, with smaller COF values at higher radii. In HPT-deformed single-phase materials, there is a typical strain-dependent saturation behavior in hardness and microstructure when exceeding a certain strain. For pure Ni, this steady state was found to occur at *ε*_eq_ ~ 8–16 [17, 21]. In the deformed IN718 sample, such saturation behavior is also observed. Backscattered electron micrographs in Fig. 11 show a homogeneous nanocrystalline microstructure above *r* = 6 mm, whereas slightly coarser grains are present at *r* = 0 mm, The observed microhardness in Fig. 1b is consistent with the microstructure. The strong grain refinement and strain hardening of the *γ* matrix likely contribute to improving the wear resistance.

In the micrographs, an irregularly shaped phase, which is brighter in contrast, is also visible. This phase was already identified in a previous work as Laves phase residues, present in the used IN718 powder, which is not sufficiently fragmented at lower strains [16]. Furthermore, the irregularly shaped Laves phase particles at the disc center may favor local failure due to stress concentrations and promote increased debris formation during sliding. Increased friction and wear in laser powder bed fusion-processed IN718 have been attributed to the presence of Laves phase [22]. At the outer radial positions, particularly at *r* = 12 mm, the Laves phase was no longer detected. Therefore, its influence is mainly relevant close to the disc center at lower strains, whereas the microstructural influence at higher radii is predominantly related to the refined and hardened *γ* matrix.

A previous in situ high-temperature XRD study showed that strengthening phases such as *γ*′ and *γ*″ are not expected under the present conditions, since they precipitate during annealing at elevated temperatures (~  600 °C), which was not applied in this study [15]. Likewise, the *γ*″ to *δ* phase transformation requires even higher temperatures and longer exposure times. In the case of MMCs, pronounced chemical interactions between the IN718 matrix and the SL particles occur only above approximately 600 °C, where metal carbide and sulfide formation can take place [15]. Such phase transformations or reaction products are therefore not expected during the present RT tests. Furthermore, nanocrystalline crystallite sizes were obtained for the TMD- and CNM-reinforced IN718 composites in the as-deformed state and remained stable up to approximately 700 °C before the onset of recrystallization. The low load and sliding speed were selected to limit frictional heating and thereby reduce the likelihood of undesired phase formation in the already complex tribosystem, such as reactions between elements of the SL particles and the IN718 matrix. Thus, at higher radii, the tribological response of the MMCs under the present conditions is governed by the refined γ matrix and by the distribution, fragmentation, and release of the embedded SL particles, rather than by the conventional phases and precipitates of IN718.

Since *N* (10 rotations) and *t* (1.5 mm) are constant for the deformed specimen, the applied strain varies only with the radial position. Thus, the simultaneous increase in strain and decrease in friction coefficient suggests a correlation between these parameters under equivalent tribological conditions. Further increases in shear strain are unlikely to result in further COF reduction, as the COF values at radial positions of 6 and 12 mm lie within their standard deviations, indicating that a steady state has been reached.

With a *µ*_EOT_~0.9–1, IN718 exhibits a high friction level during dry sliding against Si_3_N_4_ at an average sliding speed of 0.44 mm/s (Set 1), which decreases to *µ*_EOT_ ~ 0.75 at 1.13 mm/s (Set 2). This trend agrees with the observations of Zhao et al., who reported increasing COF with decreasing sliding speeds for the IN718-Si_3_N_4_ tribopair and attributed this behavior to the development of smoother, compacted oxide-rich surface layers at higher sliding speeds that reduce direct metal-ceramic contact [23]. It is known that strong adhesion can occur in ceramic–metal contacts due to chemical affinity, where metals forming stable oxides (e.g., Cu, Al, Ni) typically exhibit high friction against ceramics, whereas metals with unstable oxides (e.g., Au, Ag) show lower friction coefficients [24].

The COF values (Fig. 1) of IN718 are in good agreement with the observed wear (Fig. 2), showing a broader wear track and higher friction values with decreasing strain. This is probably linked to the lower hardness in the disc center compared to the outer radial positions, which promotes greater penetration of the harder Si_3_N_4_ spherical CB (~ 1400 HV) and thus increases the nominal contact area between the pair. The maximum Hertzian contact stress for this pairing is 577 MPa at a load of 0.5 N, and the yield strength can be estimated from the hardness divided by a Tabor factor of ~ 2.8 to approximately 2000 MPa, which is far above the present maximum Hertzian contact stress [16, 25, 26]. This indicates that the macroscopic contact conditions remain predominantly elastic. Nevertheless, localized asperity contacts can induce severe microscale plastic deformation, which drives the wear mechanisms observed. A similar relation applies to the sphere-on-flat contacts in Set 2, with maximum Hertzian contact stress of 727 MPa for Si_3_N_4_ against IN718 and 637 MPa for 100Cr6 against IN718.

### Wear-in Behavior of IN718

Despite the low normal loads and sliding speeds under ambient conditions, tribo-induced oxidation is facilitated by the nanocrystalline microstructure of IN718, which provides a high density of grain boundaries acting as diffusion pathways for oxygen and alloying elements, thereby promoting rapid oxide formation [27, 28]. Accordingly, wear of IN718 against Si_3_N_4_ is governed by oxidative and abrasive mechanisms, as confirmed in Fig. 2 by a grooved appearance of wear tracks with oxygen-enriched regions, indicating oxide layer formation. Grooves aligned with the sliding direction (Fig. 2) show sharp boundaries, which is characteristic of microcutting, while lateral pile-up indicates that microplowing is present as well. The presence of cracks within the oxide layer suggests mechanical instability under cyclic sliding, leading to repeated oxide fracture and spallation. The detached oxide fragments are either pushed out or captured within the sliding contact, where they are further fragmented into fine debris acting as third bodies and promoting abrasion, potentially transitioning from mild to severe wear conditions. This is consistent with the tribo-oxidation observations on nanocrystalline Ni by Grützmacher et al., who reported that oxidation and spallation initiate already during the first 10 cycles. At higher cycle numbers, they propose a cyclic process of oxide formation and fragmentation upon reaching a critical thickness, where the oxide becomes mechanically unstable. The resulting fragments are incorporated into the near-surface layer, while oxide regeneration occurs on newly exposed metal regions [27].

According to P.J. Blau’s models, the COF evolution of IN718 exhibits typical running-in (wear-in) behavior of unlubricated oxidized metals, characterized by an initially high wear rate (friction peak) during asperity removal, followed by surface smoothing and the formation of a debris or transfer layer [19, 20]. Initial COF fluctuations (stage 1) arise from asperity removal, followed by a short, relatively constant COF region (stage 2), which is attributed to oxide film formation, reducing direct contact of IN718 with the CB. With increasing sliding time, the COF increases again as wear debris accumulates and surface roughening progresses until a protective debris or tribolayer develops, leading to a tentative steady state friction regime (stage 3). It is known that small normal loads, as applied in this work, can prolong the wear-in (stages 1 and 2) due to slower oxide film removal and its persistence [20]. A similar COF evolution was observed for both tribopairings, IN718-Si_3_N_4_ and IN718–100Cr6, with the latter showing faster stabilization in the friction coefficient (see Fig. 5), which is likely attributed to the mutual formation of oxide layers in both IN718 and the 100Cr6 CB.

#### Influence of Counterbody Material on the Wear Behavior

**Fig. 12:**
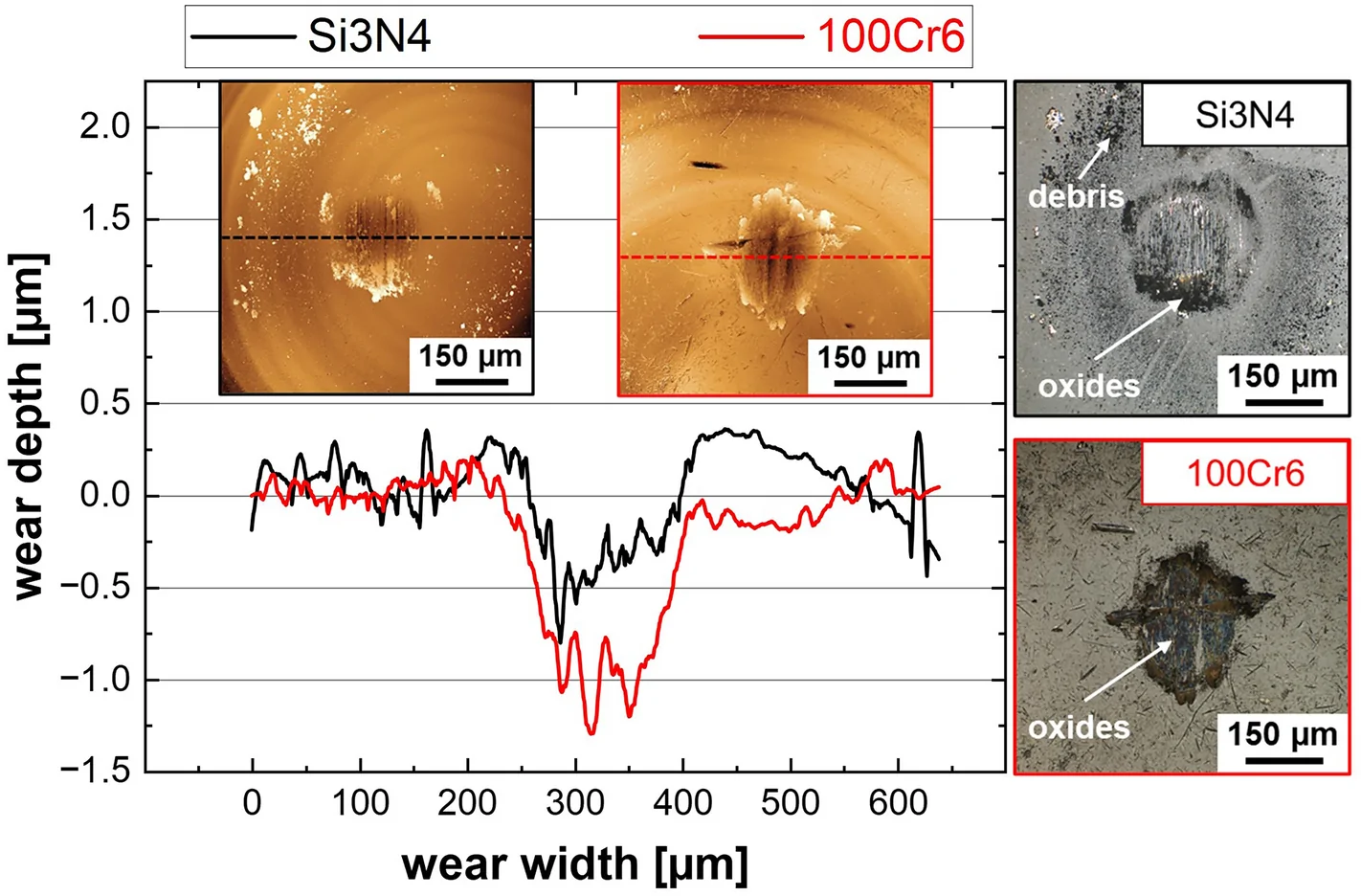
Wear track micrographs and wear profiles of Si_3_N_4_ and 100Cr6 CB tested against IN718 for 472 cycles, acquired by CLSM. The respective wear track micrographs of the IN718 counterparts are shown in Fig. 6

CB wear was analyzed for selected representative systems to support the interpretation of the dominant wear mechanisms, including the IN718 reference tested against Si_3_N_4_ and 100Cr6, and in the later Sect. 4.4.2, the 5 wt% GNP composite tested against 100Cr6 as a representative self-lubricating system. When sliding against 100Cr6, CLSM micrographs of IN718 (Fig. 6) reveal significantly deeper wear grooves compared to Si_3_N_4_, indicating more pronounced cutting wear, accompanied by higher specific wear rates (Fig. 7b). This behavior is a result of mutual wear of both IN718 and the CB, with greater wear observed on the 100Cr6 balls than on Si_3_N_4_, as shown in Fig. 12.

The Si_3_N_4_ CB exhibits an average wear depth of 0.45 µm. In contrast, the softer 100Cr6 CB shows a greater wear depth of 1.2 µm and deeper groove formation, consistent with the wear observed on the IN718 counterpart. The higher wear resistance of the Si_3_N_4_ CB is likely associated with its higher hardness compared to the 100Cr6 CB, 1400 and 770 HV, respectively. Furthermore, the oxide distribution differs significantly between the two tests. On the 100Cr6, oxides cover the entire wear scar. Conversely, on the Si_3_N_4_, oxides are concentrated at the reversal points, with debris accumulating primarily around the wear track. This suggests limited embedding of wear particles into the ceramic CB, and they are instead ejected from the contact zone. Consequently, the oxides found at the Si_3_N_4_ reversal points likely result from material transferred from the IN718. For the 100Cr6, while the oxides could also stem from IN718 transfer, they are more likely to result from oxidation of the 100Cr6 CB itself, given the extensive coverage. Notably, definitively distinguishing between IN718-derived and 100Cr6-derived oxides is challenging, as both materials form chemically similar oxides. These observations indicate that the mutual oxidation of IN718 and the 100Cr6 CB facilitates the embedding of oxide fragments at the interface, thereby exacerbating groove formation (microcutting).

### Hardening Behavior of Solid Lubricant-Reinforced IN718 Composites

In Set 1, all SL-reinforced composites (C, MoS_2_, and WS_2_) exhibit higher hardness than the IN718 reference, indicating a reinforcement effect by the embedded SL particles in addition to the strain hardening through HPT deformation. The TMD-reinforced composites show a stronger hardness increase than the C-reinforced composite (Fig. 10). Similar behavior was already observed and discussed in a previous work on the same composites processed as smaller HPT discs [15]. In the as-deformed state, IN718-C and IN718-MoS_2_ showed different hardening trends. IN718-C exhibited peak hardening, with the highest hardness at approximately 0.25 wt% C, followed by softening at higher C contents. At 5 wt% C, the hardness was lower than that of the IN718 reference. The strengthening was attributed to C particles acting as barriers for dislocation motion during deformation, thereby increasing the composite strength. In addition, C degradation may contribute to interstitial hardening of the γ matrix by released carbon [16]. The softening at higher C contents was attributed to the comparatively low hardness of C, which increasingly dominates once a certain SL fraction is exceeded. In contrast, IN718-MoS_2_ showed a continuous increase in hardness with increasing MoS_2_ content, reaching the highest hardness at 5 wt% MoS_2_. This monotonic hardening was attributed not only to the higher intrinsic hardness of MoS_2_ compared to C, but mainly to partial MoS_2_ degradation during HPT, which releases Mo into the IN718 matrix and promotes solid-solution strengthening [15].

In Set 2 (Fig. 10), the carbon-based composites (C, GNP, and fCNT) exhibit comparable or lower hardness than the corresponding IN718 reference. At the same time, the two IN718 reference materials show very similar hardness despite the different applied strains, indicating that the saturation hardness of IN718 is already reached after 2.5 HPT rotations (*ε*_eq_ ~ 73). At this strain level, the carbon-based SL particles result in softening of the IN718 MMCs, whereas the four times higher strain in Set 1 leads to hardening in the C-reinforced IN718 composite. This indicates that a certain strain is necessary to achieve a hardening effect in the composite and to overcome the lower hardness contribution of the embedded SL particles. Previous nanoindentation experiments on HPT-processed IN718-C support this assumption, showing that proper fragmentation of C agglomerates is required to achieve homogeneous hardness and hardening, with smaller SL particles being beneficial for the overall hardness of the composite [12]. The lower hardness of the fCNT-reinforced MMCs in Fig. 10 may be related to the larger particle volume fraction introduced at the same weight fraction, since fCNTs have a lower density than the layered carbon-based SL particles and can therefore contribute more strongly to the overall softening.

While the hardness values can be compared between the two specimen sets, their tribological behavior is considered separately due to the differences in tribological testing parameters. The hardness measurements help to distinguish mechanical strengthening effects from lubrication-related effects. In Set 1, hardening is observed for all conventional SL-reinforced composites, particularly for the TMD-reinforced materials. In contrast, similar or lower hardness values are observed for the carbon-based composites in Set 2. Therefore, the tribological response of Set 2 is likely attributed to lubrication-related effects (e.g., local availability of SL particles) and microstructural effects rather than to increased hardness.

### The Role of the Solid Lubricant Type, Concentration, and Counterbody Material

#### Limitations of Conventional Solid Lubricants in IN718 Composites

Unlike C-reinforced IN718, TMD-reinforced composites (Fig. 3) show no evidence of COF reduction over the entire sliding distance. On the contrary, the quasi-steady state friction of MoS_2_ and WS_2_ composites is even ~ 7–10% higher than that of unreinforced IN718, while the COF in the intermediate running-in stage of graphitic MMC is consistently ~ 0.08 lower. These differences between C and TMDs might be explained by the tribological testing environment and the structural integrity of the SL.

Raman spectroscopy measurements on IN718-C from a previous study have already shown that SPD induces a high defect density in the embedded C reinforcements, thereby reducing crystallinity. However, even after a strain exposure of *ε* ~ 540, C reinforcements are still not amorphized and remain nanocrystalline [12]. As TMD-reinforced composites were exposed to a similar strain level, a similar tendency toward defect accumulation within the MoS_2_ and WS_2_ structures cannot be excluded. Such deformation-induced structural modifications may affect interlayer shear behavior and therefore potentially influence the lubricating response of the SL reinforcements after HPT processing. On the other hand, all MMCs in this work were tested under ambient conditions (RT at ~ 40% humidity). It is known that TMD coatings, such as MoS_2_ and WS_2_, exhibit superior lubricity when sliding in vacuum (COF ~ 0.05–0.005 at RT) [29]. However, they show increasing friction coefficients (COF ~ 0.2–0.4 at RT) and shorter wear life when sliding in humid air due to the reaction of the basal plane edges with moisture and oxygen, forming MoO_3_ and WO_3_. In contrast, C coatings show the opposite behavior since adsorbed water or other condensed vapors passivates dangling bonds on reactive edge sites, which reduces interlayer adhesion, and thereby promotes easier basal plane sliding compared to vacuum conditions, resulting in a COF ~ 0.1–0.3 for ambient conditions [10, 29, 30]. This fundamentally limits the application of TMD-reinforced MMCs in standard ambient environments.

**Fig. 13:**
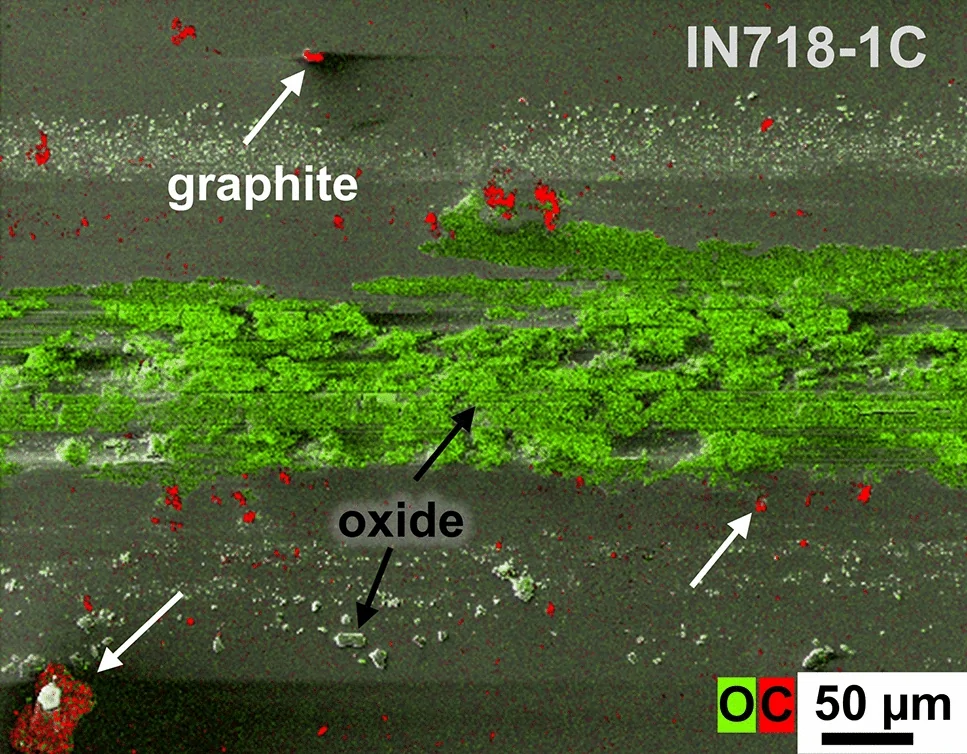
Overlay of oxygen and carbon EDS maps with the SE micrograph of the wear track of IN718-1C tested against Si_3_N_4_. The specimen corresponds to Set 1

At a fraction of 1 wt% of conventional SL, the friction and wear levels are comparable to, or even higher than, those of unreinforced IN718, and far higher than the aforementioned COF ranges when C, MoS_2_, and WS_2_ are applied as coatings. This suggests that the 1 wt% SL fraction is too low to release sufficient SL particles to form a sustainable transfer layer during sliding, particularly given that the SL reinforcements were already heavily strained and their lubricity might be diminished. This assumption is also supported by previous quantitative particle analysis of IN718-1C processed by colloidal mixing and HPT, where a mean SL particle size of approximately 0.85 µm, a median lateral particle spacing of 0.8 µm, and a homogeneity factor of ~ 80% were reported [12]. Considering the Hertzian contact radius of ~ 20–25 µm for the present sphere-on-flat contact at loads of 0.5–1 N, the probability that C particles are present within the nominal contact area is high. Therefore, the limited lubricating effect is likely not caused by a lack of C particles in the contact area, but rather by reduced lubricity upon SPD and/or insufficient retention of a stable tribolayer. This interpretation is further supported by the EDS maps of the IN718-1C wear track in Fig. 13, which show pronounced oxygen enrichment despite the presence of carbon and therefore suggest that the low SL fraction is insufficient to counteract tribo-induced oxidation. As discussed before, the nanocrystalline microstructure of IN718 promotes rapid oxide formation during sliding, which may progress faster than the development of a lubricating film. Therefore, the SL particles are covered by the oxide layer, hindering their further release. Furthermore, several C particles (indicated by arrows) are ejected from the contact together with oxide fragments, indicating additional material removal as debris during the sliding. This probably contributes to the higher specific wear rate observed in the unlubricated graphitic systems (Figs. 3b and 7), which can be up to one order of magnitude higher (*W*_*S*_ ~ 10^–7^ mm^3^ N^−1^ mm^−1^), than in the IN718 reference, indicating a transition from mild to severe wear [31]. While the TMD-reinforced IN718 composites show higher friction than the IN718 reference, the opposite trend is observed for the specific wear rate in Fig. 3b. This indicates that COF and wear rate are not governed by the same dominant mechanism in these systems. The lower wear rates of the TMD-reinforced composites are likely related to their higher hardness (Fig. 10), which improves the resistance against material removal during sliding. In addition, the TMD-reinforced IN718 MMCs were previously discussed to exhibit more homogeneous microstructures than the carbon-based SL composites, which is also reflected by their smaller standard deviations in microhardness (Fig. 10) [15]. In contrast, the C-reinforced composite exhibits a higher wear rate despite its higher hardness relative to the IN718 reference material. This suggests that C particle removal, together with oxide fragments, counteracts the strengthening effect and promotes additional material loss during sliding.

Overall, the TMD- and C-reinforced IN718 composites exhibit wear characteristics similar to unreinforced IN718 (see Fig. 4), where tribo-induced oxide layers form, fracture, and act as abrasive debris. Additional debris may be generated, likely due to the pull-out of SL particles together with oxide fragments when sliding against Si_3_N_4_, which is consistent with the wear tracks in Fig. 6. Despite the expected presence of C particles within the nominal contact area, the limited amount of SL appears insufficient to form a stable lubricating transfer layer to counteract these oxidative processes, although the incorporation of SL particles may locally reduce interfacial shear strength and contribute to the initial more pronounced COF fluctuations within the first cycles (Figs. 3 and 5). Unlike C, the investigated TMD reinforcements did not exhibit any indications of solid lubrication against Si_3_N_4_ under ambient conditions. Thus, further investigations focused on more promising carbon-based SL (C, GNPs, fCNT) in an independent but complementary specimen set (Set 2), using different tribological testing parameters, various SL concentrations, and alternative CB materials. Therefore, the following results are evaluated within Set 2 and are not directly compared with those in Set 1.

#### Self-Lubrication Characteristics of IN718 Composites Reinforced with Carbon Nanomaterials

The wear behavior of CNT-reinforced composites agrees well with the friction curves in Fig. 5. Composites exhibiting reduced COF levels, particularly the fCNT-containing materials, also show reduced wear width, as evident in the CLSM micrographs in Fig. 6. For the 5GNP and 5fCNT composites tested against the 100Cr6 CB, hardly any wear is visible at this magnification, while only a few isolated scars are observed in the magnified CLSM micrographs in Supplementary Fig. S3. The smooth COF evolutions, showing steady state friction values near 0.1, and no prominent wear, are characteristics of self-lubricating behavior. It should be noted that although the IN718-5fCNT composite tested against 100Cr6 was exposed to fewer cycles (261), the COF had already reached a stable friction level after the running-in stage and remained nearly constant throughout the recorded test duration. Therefore, no pronounced change in friction behavior would be expected during the remaining cycles.

**Fig. 14:**
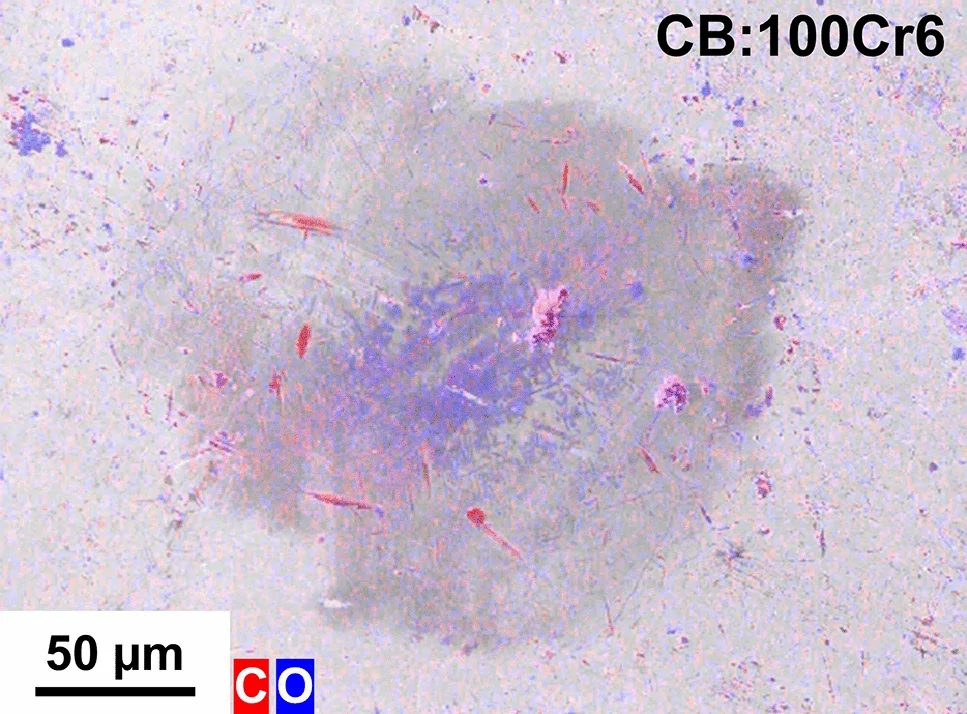
EDS overlay (carbon and oxygen maps) with the SE micrograph of the 100Cr6 CB’s wear track from sliding against IN718-5GNP composite

Wear analysis of the 100Cr6 CB used against the 5GNP composite (Fig. 14) exhibits mild wear, even though initial oxidation is observed. Furthermore, EDS maps reveal an accumulation of carbon at the CB contact zone, indicating the formation of a lubricious carbon-rich tribolayer.

In contrast to the 100Cr6 CBs tested against systems without clear self-lubricating behavior, no abrasion is observed here. This supports the assumption that the carbon-rich tribolayer and transfer layer reduce direct metal–metal contact and thereby suppress the previously discussed mutual oxidative-abrasive wear mechanisms. This likely contributes to the observed ~ 5–6 times lower *µ*_EOT_ = 0.14 and approximately one order of magnitude smaller wear rate (~ 10^–9^ mm^3^ N^−1^ mm^−1^).

Compared to the IN718 reference and the self-lubricating composites, materials exhibiting higher COF levels show a higher density of particle-shaped pits within the wear track, as evident in the height profile images in Fig. 6. This indicates higher debris formation through particle pull-out as already described for IN718-C, which results in higher friction instabilities in the initial wear-in and leads subsequently to increased friction and wear. As the carbon-based composites in Set 2 show comparable or lower hardness than the corresponding IN718 reference (Fig. 10), hardness differences are not expected to dominate the tribological response in this specimen set. When sliding against the 100Cr6 CB instead of the ceramic one, increased cutting (see Fig. 6) is observed for the composites, with friction levels similar to those of the IN718 reference. The mutual oxidation of the MMC and 100Cr6 CB promotes enhanced abrasive cutting, as already discussed in Sect. 4.2.

The running-in behavior of unlubricated C composites is mostly consistent with unlubricated GNP composites (see Fig. 5), both following either the friction wear-in curve shapes of unlubricated oxidized metals or metal on C contacts, as described by Blau et al. [19, 20]. The metal-on-graphite curve shape is indicated by a decreased COF within the first cycles due to the creation of a thin film, followed by a rise in friction through debris formation or material transfer. The IN718-fCNT composites follow the wear-in curve shape of boundary-lubricated metals, showing a friction peak at the beginning as the wear rate is high due to asperity removal, followed by a constant and low COF. However, at a fCNT fraction of 1 wt%, the COF is higher, and the evolution is less smooth at 5 wt%, which is an indicator for unstable lubrication through increased debris formation by metal oxidation, particularly when sliding against Si_3_N_4_. The faster friction stabilization when sliding against the 100Cr6 is likely associated with the enhanced bonding of the CNMs at the counter surface. In this context, MacLucas et al. showed that for CNT-coated iron, the CNT structure progressively degrades during sliding, accompanied by tribochemical reactions that form an interfacial oxide/carbide layer. This layer covalently anchors a CNT-derived tribofilm to the metallic surface, thereby shifting the shear zone toward the carbonaceous tribofilm–CB interface, resulting in solid lubrication. Moreover, it was shown that starvation of the SL within the wear track can occur when the CB pushes tribomaterial toward the track’s reversal points during reciprocating sliding, which leads to increased oxidation in the contact zone and, consequently, higher friction and wear [32].

**Fig. 15:**
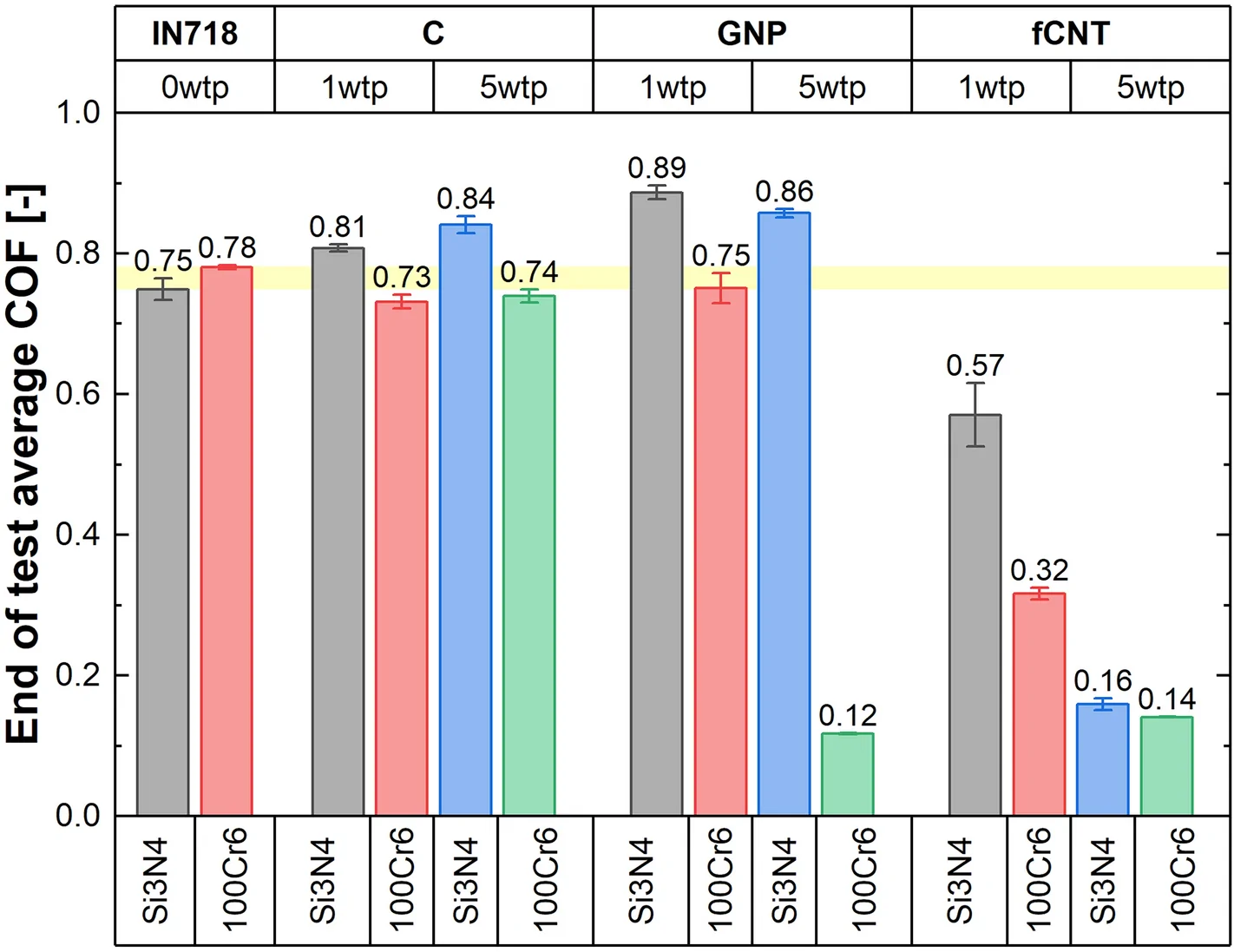
End-of-test average COF (*µ*_EOT_) values of IN718 reinforced with either 1 or 5 wt% of C, GNP, or fCNT, tested against spherical Si_3_N_4_ or 100Cr6 CBs. The µ_EOT_ values were calculated from the last 20% of the recorded COF data shown in Fig. 5, while the error bars represent the corresponding standard deviation within each individual measurement. The horizontal yellow line is a guideline to the eye representing the µ_EOT_ range of the IN718 reference

In general, the carbon-based composites exhibit variations in COF levels, which are summarized as µ_EOT_ values in Fig. 15 for improved comparability. The influence of SL type (C, GNP, and fCNT), concentration, and CB material is presented, while the yellow horizontal line indicates the COF range of the IN718 reference tested against Si_3_N_4_ and 100Cr6. Among the investigated MMCs, only IN718-fCNT composites show a pronounced friction reduction across all tested concentrations and independent of the CB material, with µ_EOT_ decreasing with increasing SL fraction. Similar trends of decreasing friction with increasing SL content have been reported for MMCs with various matrices, although friction and wear may increase once the optimum SL content is exceeded [11]. This may also affect tribo-oxidation behavior.

C- and GNP-reinforced IN718 exhibit similar or slightly higher COF levels compared to unreinforced IN718, except for the 5GNP composite tested against the 100Cr6 CB (*µ*_EOT_ = 0.12). In general, lower friction values were observed when sliding against the 100Cr6 CB, which may be attributed to mutual oxidation of the tribopair, reducing direct metal–metal contact. Conversely, higher specific wear rates were generally observed against 100Cr6, correlating with the more pronounced microcutting, likely attributed to additional debris generation through the mutual oxidation. In addition, improved interfacial bonding of CNMs with the 100Cr6 ball compared to the ceramic counterpart, as indicated in Fig. 12, may promote localized lubrication and the formation of a lubricating transfer layer.

The IN718-5CNT composite exhibits *µ*_EOT_ values of from 0.14 to 0.16 for both CB materials, corresponding to an approximately fivefold friction reduction relative to the IN718 reference and lying within the range reported for CNT coatings on metallic substrates [32]. This indicates that, despite HPT-induced structural degradation of CNTs, effective lubrication can still be achieved in the MMCs. Aristizabal et al. investigated structural defects of multi-walled CNTs (as used in this work) embedded in a Ni matrix exposed to HPT deformation [13]. The study showed that maximum CNT damage occurs at early deformation stages, before microstructural saturation of the MMC is reached, and that this transition appears at lower strains with increasing CNT content. Despite pronounced structural degradation across different CNT concentrations and equivalent strains up to 375, Raman measurements implied no amorphization. This was attributed to partial shielding of the CNTs by the metallic matrix, which absorbs a part of the deformation-induced strain energy. Furthermore, it was proposed that CNTs follow an amorphization trajectory, where cylindrically shaped nanotubes progressively fragment into flattened graphite/graphene-like structures before undergoing amorphization. Consequently, even degraded CNTs may retain sp^2^-bonded graphitic structures over a wide deformation range. This behavior could explain the improved tribological response of the HPT-deformed IN718-fCNT composites in the present study. Compared to IN718-C or IN718-GNP MMCs, fCNTs appear to maintain their lubricating capability longer despite structural alterations caused by SPD (as applied for all systems) and additional induced damage by prior CNT functionalization [33].

Overall, fCNT reinforcements show the most promising performance among the tested SL, providing significant reductions in friction and wear in the nanocrystalline IN718 composites across all concentrations and CB materials. Particularly, IN718-5fCNT exhibits self-lubricating characteristics with smooth running-in behavior and mild (oxidative) wear. In contrast, the other tested SL reinforcements (MoS_2_, WS_2_, C, and GNPs) were shown to be insufficient to counteract the rapid tribo-oxidation of the nanocrystalline IN718 matrix, which is accompanied by abrasive wear due to oxide debris. As a result, only limited improvements (or even deterioration) were predominantly achieved in either friction or wear, but not both simultaneously, depending on SL concentration and CB material. The only exception is the 5GNP composite, which exhibits self-lubricating characteristics comparable to those of the 5fCNT composite, but only when sliding against 100Cr6.

## Conclusions

The tribological behavior of severely strained and nanocrystalline IN718 and its composites reinforced with various SL particles was investigated under ambient conditions. Ball-on-disc tests were performed against Si_3_N_4_ CBs under linear reciprocating motion, while carbon-based composites were additionally tested against 100Cr6. Particular emphasis was placed on the running-in behavior, since this early sliding stage provides insight into whether the different SL types and concentrations can establish self-lubrication in the MMCs. The following conclusions can be drawn, which are limited to the investigated processing and testing conditions, including nominal elastic contact conditions, low sliding speeds, and small sliding distances:

- The radial testing position on HPT-deformed IN718 discs is important, as COF evolution is strain-dependent and reaches a saturation-like behavior with increasing deformation, consistent with microstructure and hardness saturation. The nanocrystalline microstructure of IN718 promotes rapid tribo-oxidation, with fractured oxide layer particles acting as third-body abrasives. Nevertheless, mild wear was observed (*W*_*S*_ ~ 10^–8^ mm^3^ N^−1^ mm^−1^).
- Conventional SL (MoS_2_, WS_2_, and C) did not establish a lubricating film under the tested conditions, resulting in oxidation- and abrasion-dominated wear. Consequently, improvement in tribological performance was limited or, in some cases, deteriorated (e.g., IN718-5C with *W*_*S*_ ~ 10^–7^ mm^3^ N^−1^ mm^−1^).
- GNPs showed similar behavior except at 5 wt% and against 100Cr6, where a COF ~ 0.12 and *W*_*S*_ ~ 10^–9^ mm^3^ N^−1^ mm^−1^ were obtained, suggesting that for GNPs, both a suitable CB material and sufficient SL appear to be important to form an effective lubricating tribofilm.
- For tribo-systems without clear self-lubrication, sliding against 100Cr6 results in more pronounced microcutting and higher wear rates compared to Si_3_N_4_ but lower friction coefficients. This behavior is likely attributed to enhanced debris generation and mutual tribo-oxidation of the CB and composite surface.
- Among the tested SL reinforcements, fCNT demonstrated the most consistent reduction in both friction (up to ~ 5 ×) and wear (up to one order of magnitude) across the tested SL fractions and CB materials, by effectively suppressing tribo-induced oxidative-abrasive wear mechanisms by forming a carbonaceous self-lubricating tribolayer. A COF of ~ 0.14 and *W*_*S*_ of ~ 10^–9^ mm^3^ N^−1^ mm^−1^ were obtained for the IN718-5fCNT composite.

Overall, despite the structural alteration of SL introduced by SPD, fCNT-reinforced IN718 exhibited the most promising tribological performance under the conditions investigated, highlighting its potential for advanced, more sustainable applications of Ni-based superalloys. Future work should investigate their long-term lubricity under higher loads and speeds, as well as the tribological response under vacuum and elevated temperatures, where oxidation and intrinsic lubricity are expected to change.

## Supplementary Information

Below is the link to the electronic supplementary material.

Supplementary file1 (PDF 398 KB)

## Data Availability

A Supplementary data file is appended to this article. The data used in this study are openly available in the Zenodo repository at https://doi.org/10.5281/zenodo.19915797.
