# Supplementary material for: Optimizing the Tribological Behavior of Nanocrystalline Inconel 718 by Solid Lubricant Reinforcements
Source: Tribol Lett. 2026 Sep 4;74(4):104. doi: 10.1007/s11249-026-02198-x (PMC13545129; doi:10.1007/s11249-026-02198-x)
Supplement: Supplementary file 1 [file 11249_2026_2198_MOESM1_ESM.pdf]

Supplementary Information for

"Optimizing the tribological behavior of nanocrystalline Inconel 718 by solid lubricant reinforcements"

Manoel Kasalo[a*], U. Pranav Nayak[b], Timothy MacLucas[b], Sebastian Suarez[b], Andrea Bachmaier[a]

[a] *Erich Schmid Institute of Materials Science of the Austrian Academy of Sciences, 8700 Leoben, Austria*
[b] *Chair of Functional Materials, Saarland University, Campus D3.3, 66123 Saarbrücken, Germany*

**Corresponding author e-mail address: manoel.kasalo@oeaw.ac.at*

Supplementary Methodology

Colloidal mixing details:

Each solid-lubricant powder was dispersed in ethylene glycol at a ratio of 0.5 mg mL^{-1}. To promote the formation of a stable suspension, the graphite-containing mixtures were first subjected to high-shear mixing at 7500 rpm for 10 min using an IKA T 25 disperser, followed by ultrasonication for 10 min in a Bandelin Sonorex DT 510 bath to reduce agglomerate size. Subsequently, Inconel 718 powder was added to the suspension and homogenized by an additional 10 min of high-shear mixing at 7500 rpm. The solvent was then removed by drying in a convection oven at 150 °C for several hours. After gentle mortaring and a secondary drying step, the IN718 + solid lubricant powder blend was obtained and prepared for subsequent HPT processing.
Functionalization was performed on the multi-walled carbon nanotube powder in an HNO_3/H_2SO_4 mixture for 72 h at 80°C to improve the interfacial compatibility between SL and IN718 powder, following the procedure reported in reference [MacLucas, 2023, https://doi.org/10.1016/j.cartre.2023.100265].

Used Powder Materials:

Table S1. Matrix and solid lubricant powder materials used for composite fabrication, including supplier, morphology, size, and purity. TMD = transition metal dichalcogenides, CNM = carbon nanomaterial, C = graphite, GNP = graphene nanopowder, CNT = carbon nanotubes.

**Type (role)**	**Material**	**Fractions [wt%]**	**Size [µm]**	**Purity [%]**	**Supplier**	**Morphology**
Ni-supperalloy (matrix)	IN718	Bal.	< 45	-	GoodFellow	Spherical
TMD (reinforcement)	MoS_2	1	< 44	99.8	Alfa Aesar	Platelets
TMD (reinforcement)	WS_2	1	< 44	99	Alfa Aesar	Platelets
CNM (reinforcement)	C	1 and 5	< 75	99.997	GoodFellow	Layered flakes
CNM (reinforcement)	GNP	1 and 5	Lateral: 2-8<br>Height: 0.003	> 98	Graphene Supermarket	Platelets
CNM (reinforcement)	CNT	1 and 5	Ø: 0.05-0.085<br>Length: 10-15	> 94	Graphene Supermarket	Tubular

The chemical compositions of the γ matrix and Laves phase in the HPT-processed IN718 were determined and discussed in detail in our previous work. The γ matrix has a mean elemental composition (given in wt%) of 51.8 ± 0.5 Ni, 19.4 ± 0.2 wt.% Cr, 17.9 ± 0.1 Fe, 4.7 ± 0.2 Nb + Ta, 3.4 ± 0.3 Mo, 0.8 ± 0.3 Al, 1.0 Ti, 1.0 Co, and 0.6 ± 0.3 minor elements, including C, Mn, Si, and Cu. The Laves phase is composed of 41.0 Ni, 14.1 Fe, 17.8 Cr, 5.9 Ti, 2.4 Mo, and 18.8 Nb (values given in at%). This composition is consistent with the A_2B-type Laves phase structure, commonly expressed as $(Ni, Cr, Fe)_2(Nb, Mo, Ti)$ [Kasalo et al., https://doi.org/10.1016/j.msea.2025.148142].

<u>Selection of HPT parameters and microstructural saturation</u>

The applied pressure of 5 GPa was selected to enable continuous plastic deformation of the high-strength IN718-based composites and to reduce slipping between the specimen and the anvils. In HPT processing, pressures of several GPa are typically required to generate sufficient friction at the specimen-anvil interface and to achieve material flow, while the applied pressure must remain within the mechanical limits of the working tools (anvils). This is particularly important for the present IN718 composites, since the incorporated solid lubricant particles can promote slipping during deformation. The low rotational speed of 0.07 min^{-1} was chosen to keep the deformation mechanically stable and to avoid excessive intrinsic heating or machine torque overload.

The formation of a saturated microstructure during HPT depends on whether a single-phase, multiphase, or composite material is processed [Pippan et al., https://doi.org/10.1002/adem.200600133]. As summarized by Pippan et al., saturation in single-phase materials results from a balance between deformation-induced refinement and recovery/restoration processes. With increasing shear strain, dislocations accumulate, the original grains are subdivided into cells and subgrains, the misorientation between neighboring structural elements increases, and an ultrafine-grained or nanocrystalline microstructure forms. Above a critical strain, further deformation does not lead to a significant further decrease in grain or crystallite size. For multiphase materials and metal matrix composites, the saturation behavior is more complex. In addition to matrix grain refinement, severe plastic deformation can cause homogenization of particle distributions, particle fragmentation, phase dissolution, or mechanical alloying, depending on the hardness difference, volume fraction, deformability, and stability of the individual phases [Pippan et al., https://doi.org/10.1002/adem.200600133].

In the present IN718–SL composites, the applied strain was therefore required not only to refine the IN718 matrix, but also to promote co-deformation, fragmentation, and distribution of the embedded SL particles. However, “microstructural saturation” in the manuscript primarily refers to the saturation in refinement of the IN718 γ matrix. The fragmentation and distribution of the SL particles are considered as continuous processes. The saturation in refinement of the nanocrystalline γ matrix is reached within the first HPT rotations, which is promoted by the high number of alloying elements in IN718 and further supported by the initially present hard Laves phase. This phase is fragmented down to the nanoscale or even dissolved during deformation. Increasing the deformation temperature results in higher fractions of undesired Laves Phase, thus, the composites were processed at room temperature. These effects are described in a previous work [Kasalo et al., https://doi.org/10.1016/j.msea.2025.148142].

Supplementary Figures

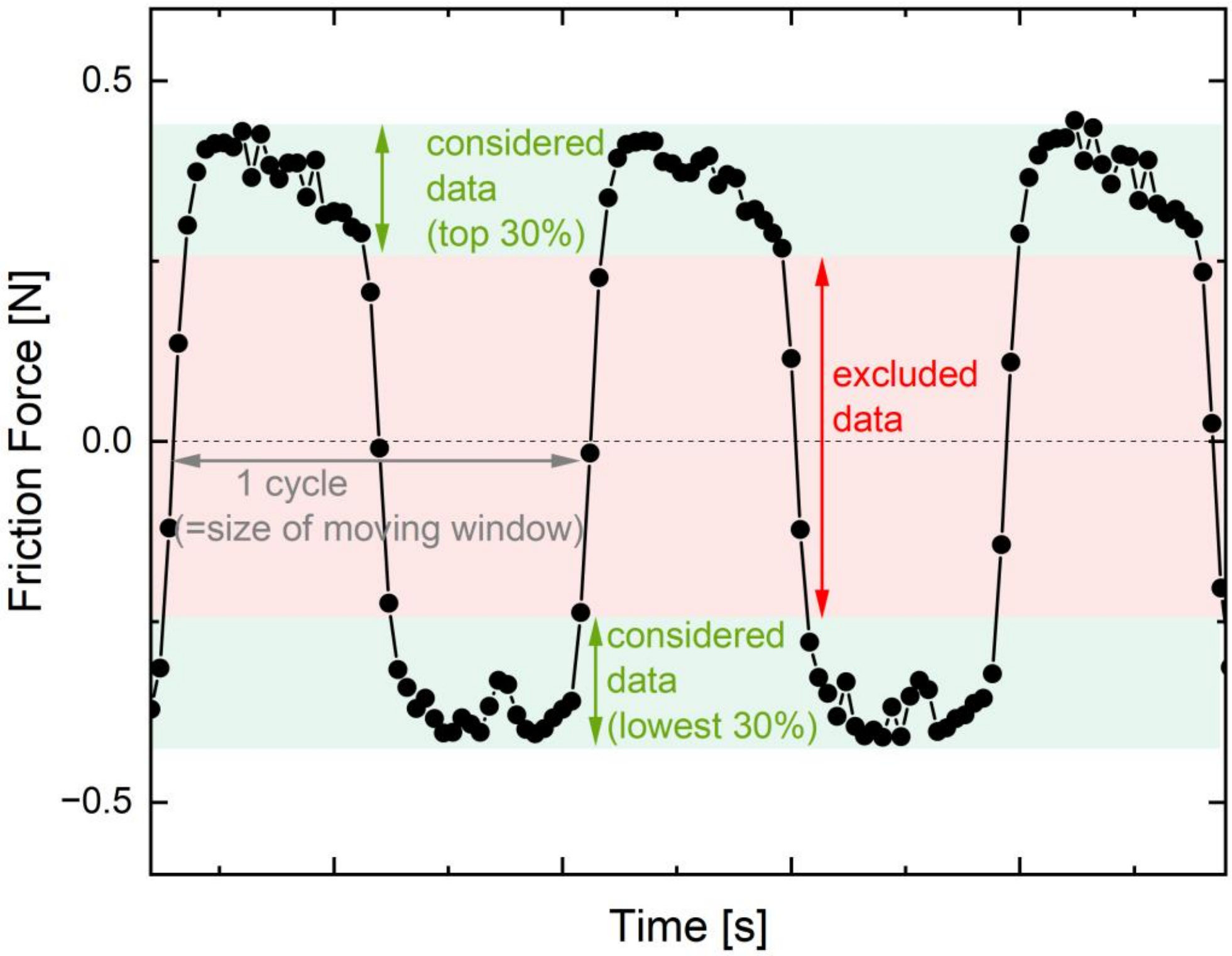

Fig. S1. Representative friction force signal during linear reciprocating sliding. The data in the vicinity of the velocity reversal points (red-shaded regions) were excluded from analysis, as the sliding velocity approaches zero in these regions, leading to transient friction force fluctuations associated with the change in direction of motion that are not representative of steady sliding. The remaining data (green-shaded regions) were considered in the analysis to calculate the COF. The considered data was processed (filtered) using a cycle-based peak filter in the proprietary Bruker UMT Tribolab software, in which the top and bottom 30% of values were averaged within a moving window corresponding to one reciprocating cycle.

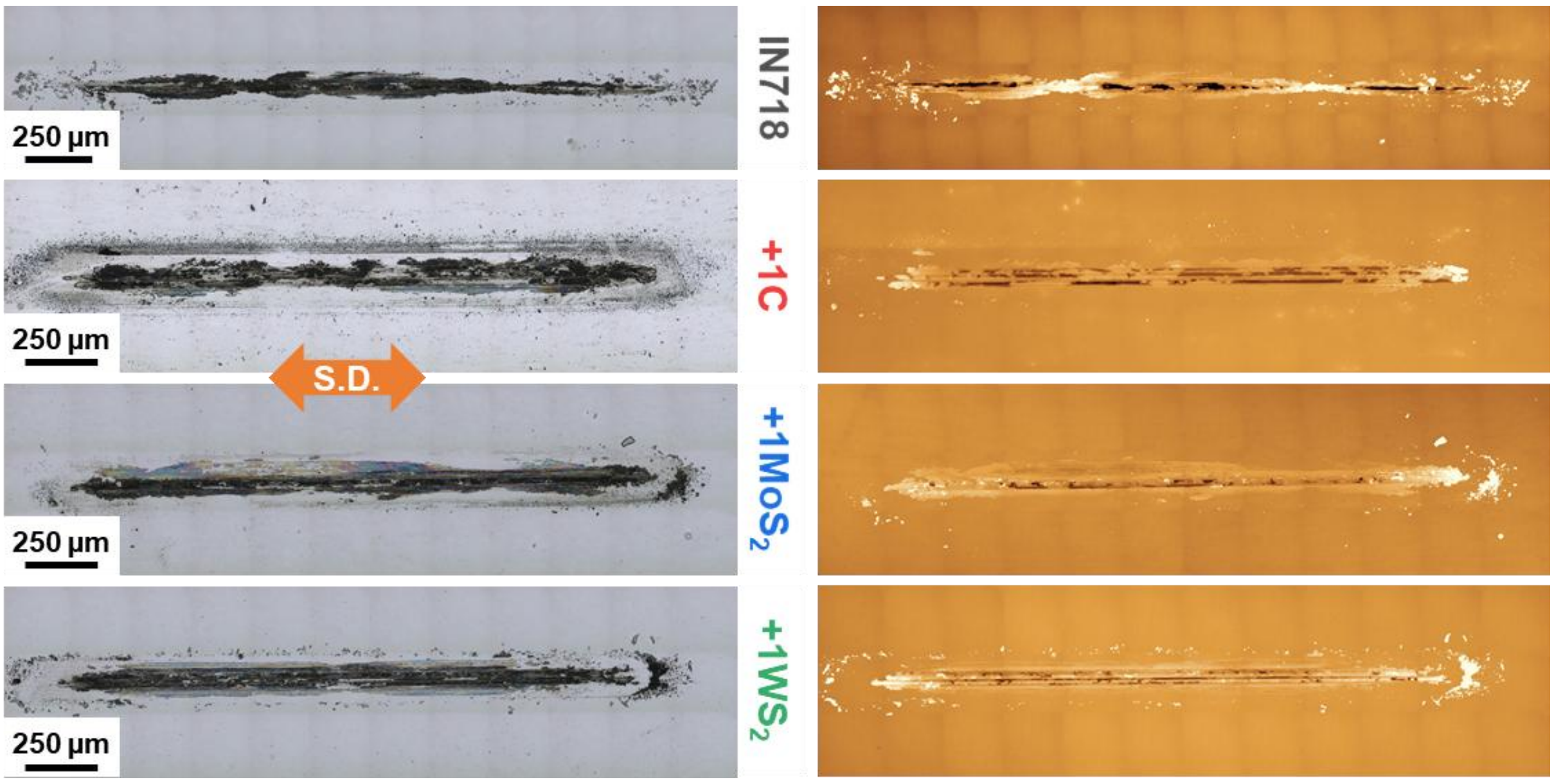

Fig. S2. Confocal laser scanning microscopy micrographs of the wear track of IN718 and IN718 composites reinforced with 1 wt% of either graphite (C), MoS_2, or WS_2 (Set 1). The composites were subjected to 264 cycles against a Si_3N_4 ceramic ball, corresponding to the friction curves in Fig. 3. The colored CLSM micrographs (right) represent the height profiles of the optical micrographs (left), with lower intensities indicating lower surface heights. The sliding direction (S.D.) is indicated by the arrow.

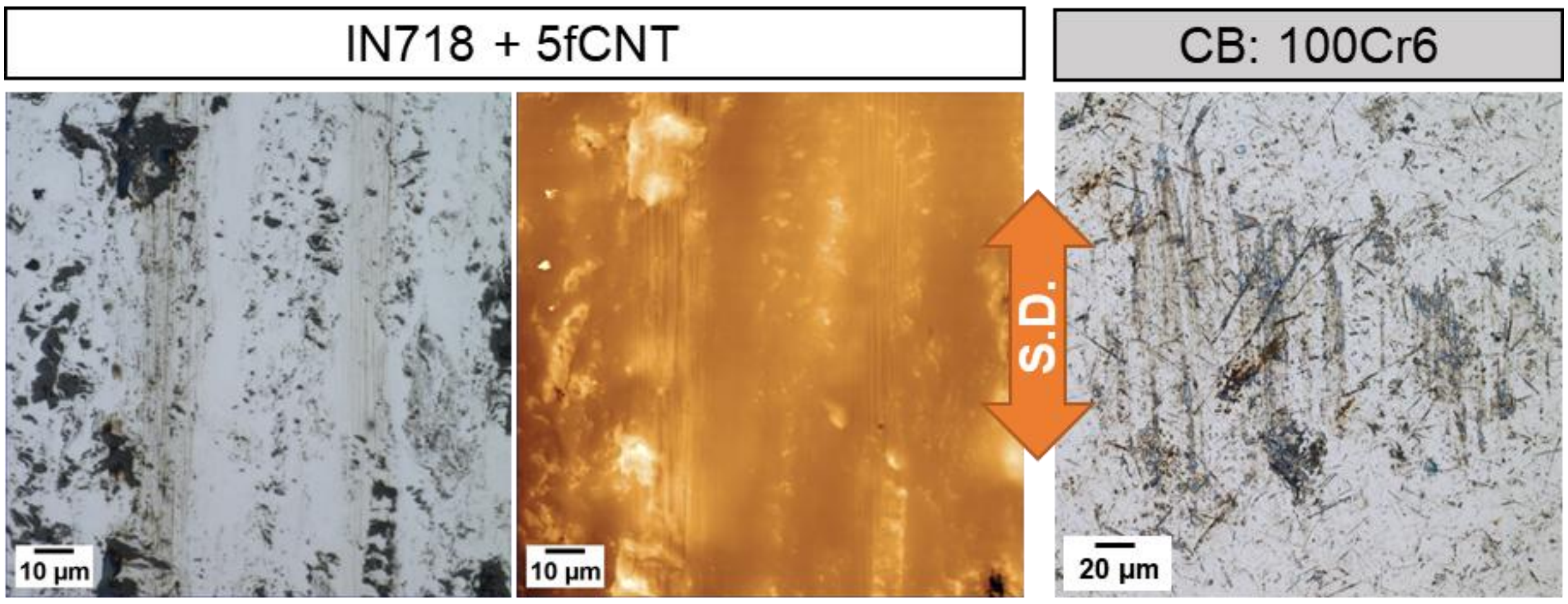

Fig. S3. Confocal laser scanning micrographs of the wear scars formed on IN718-5fCNT (left and middle) and the corresponding 100Cr6 steel counterbody (right). The two micrographs on the left present a higher-magnification view of the IN718-5fCNT wear scar shown in Fig. 6(d). The sliding direction (S.D.) is indicated by the arrow.
